# Tectonic architecture of the northern Dora-Maira Massif (Western Alps, Italy): field and geochronological data

**DOI:** 10.1186/s00015-024-00459-2

**Published:** 2024-04-22

**Authors:** Francesco Nosenzo, Paola Manzotti, Mikaela Krona, Michel Ballèvre, Marc Poujol

**Affiliations:** 1 Department of Geological Sciences, Stockholm University, 106 91 Stockholm, Sweden; 2 Univ. Rennes, CNRS, Géosciences Rennes-UMR 6118, 35000 Rennes, France; 3 Department of Geosciences, University of Padova, 35122 Padova, Italy

**Keywords:** Pinerolo Unit, Germanasca Valley, Tectonic stack, U–Pb age, Zircon, Alps

## Abstract

High-pressure and ultra-high-pressure metamorphic terrains display an internal architecture consisting of a pile (or stack) of several coherent tectonic thrust sheets or units. Their identification is fundamental for understanding the scale and mechanisms active during subduction and exhumation of these crustal slices. This study investigates the geometry of the northern Dora-Maira Massif and the kinematics of the major tectonic boundaries, combining field and geochronological data. The tectonic stack of the northern Dora-Maira Massif comprises the following units. The lowermost unit (the Pinerolo Unit) is mainly characterized by Upper Carboniferous fluvio-lacustrine (meta-)sediments. The Pinerolo unit is overthrust by a pre-Carboniferous basement. The latter is subdivided in two tectonic units (the Chasteiran and Muret Units) with different Alpine metamorphism (ultra-high-pressure and high-pressure, respectively). The pre-Carboniferous basement of the Muret Unit is thicker than previously thought for two main reasons. Firstly, some paragneisses, traditionally assumed to be Carboniferous and/or Permian in age, display detrital zircon ages indicating a main source at about 600 Ma. Secondly, three samples of the Granero Orthogneiss, previously assumed to be a Permian intrusive body, have provided zircon U–Pb ages of 447 ± 1 Ma, 456 ± 2 Ma and 440 ± 2 Ma, indicating a late Ordovician or early Silurian age for the protoliths. The uppermost unit (the Serre Unit) comprises porphyritic (meta-) volcanic and volcaniclastic rocks dated to the Permian (271 ± 2 Ma), on top of which remnants of the Mesozoic cover is preserved. Detailed mapping of an area about 140 km^2^ shows that (i) the ultra-high pressure Chasteiran Unit is localized at the boundary between the Pinerolo and Muret Units, (ii) the Granero Orthogneiss may be considered as the mylonitic sole of the Muret Unit, characterized by a top-to-W sense of shear, and (iii) the contact between the Muret and Serre Units displays ductile-to brittle structures (La Fracho Shear Zone), indicating a top-to-the-NW displacement of the hangingwall with respect to the footwall. A final episode of brittle faulting, cutting across the nappe stack (the Trossieri Fault), indicates an extensional stage in the core of the Alpine belt, as previously documented in more external zones. This work provides a necessary and robust basis before an accurate discussion of processes acting during continental subduction of the Dora-Maira Massif may be understood.

**Supplementary Information:**

The online version contains supplementary material available at 10.1186/s00015-024-00459-2.

## Introduction

Subduction of continental crust is not only testified by the exhumed high-pressure (*HP*) and ultra-high-pressure (*UHP*) continental metamorphic rocks (e.g. Chopin, 1984; Coleman and Wang, 1995; Carswell and Compagnoni, 2003; Gilotti, 2013; Brown, 2023), but also observed as an ongoing process below active orogens (Schneider et al., 2013). During continental subduction a pre-existing crust is reworked. Several coherent volumes of crustal material, bounded by tectonic discontinuities, are buried independently from one another, and can reach different depths. The different volumes may consist of different types of pre-existing material, such as an old basement already metamorphosed during a former orogenic cycle (polycyclic basement), or younger magmatic and sedimentary products (monocyclic rocks). During exhumation, the different volumes are stacked on top of each other. As a result, the internal architecture of *HP*–*UHP* terranes often consists of a pile of two or more tectonic units that differ in their pre-subduction history and/or their subduction/exhumation history. Examples include the Cyclades in Greece (Forster and Lister, 2005; Grasemann et al., 2018), the northwestern Himalayan belt in Ladakh (Tso Morari: Steck et al., 1998; Epard and Steck, 2008), and the Dabie Sulu in China (Liu et al., 2004; Xu et al., 2006). In the Western Alps (for a review, see Manzotti and Ballèvre 2023), stacking of thin slices of continental material with different *P*–*T* histories has been also described in the internal zones, like the Dora-Maira (e.g. Wheeler, 1991; Henry et al., 1993; Groppo et al., 2019) and Gran Paradiso Massifs (Compagnoni et al., 1974; Le Bayon and Ballèvre, 2006; Manzotti et al., 2015). In some cases, the age of the peak metamorphism may be diachronous across the tectonic stack, indicating protracted subduction of different crustal blocks (Xu et al., 2006; Liu et al., 2009; Bonnet et al., 2022; Glodny and Ring, 2022). In other cases, peak pressure has been achieved at a similar age, before stacking of the different slices (Manzotti et al., 2018).

Several criteria can be used when attempting to differentiate tectonic units in *HP–UHP* continental terranes. Tectonic units may differ in (i) the nature and age of their protoliths and (ii) their metamorphic history in terms of *P–T* conditions and age. Lithological contrasts and *P–T* discontinuities in the structural pile may indicate the location of tectonic boundaries (e.g. Törnebohm, 1888), which would be associated with strain localization. Because analytical studies can only be performed on a few selected samples (e.g. age or *P–T* determination), laboratory-based criteria must be used in conjunction with a field-based criterion in order to allow extrapolation of the results from the sample scale (centrimetre to decimetre) to the orogenic scale (kilometre to decakilometre). In this study, we combine fieldwork (i.e. geometric and kinematic analysis) and U–Pb geochronology (i.e. nature and age of the protoliths) in order to structurally characterize the main tectonic contacts in the northern Dora-Maira Massif and to better understand the tectonic architecture of this *HP–UHP* complex. We provide a new interpretation of the northern Dora-Maira Massif that allows an accurate definition of the coherent slices involved during nappe stacking.

## Geological setting

**Fig. 1:**
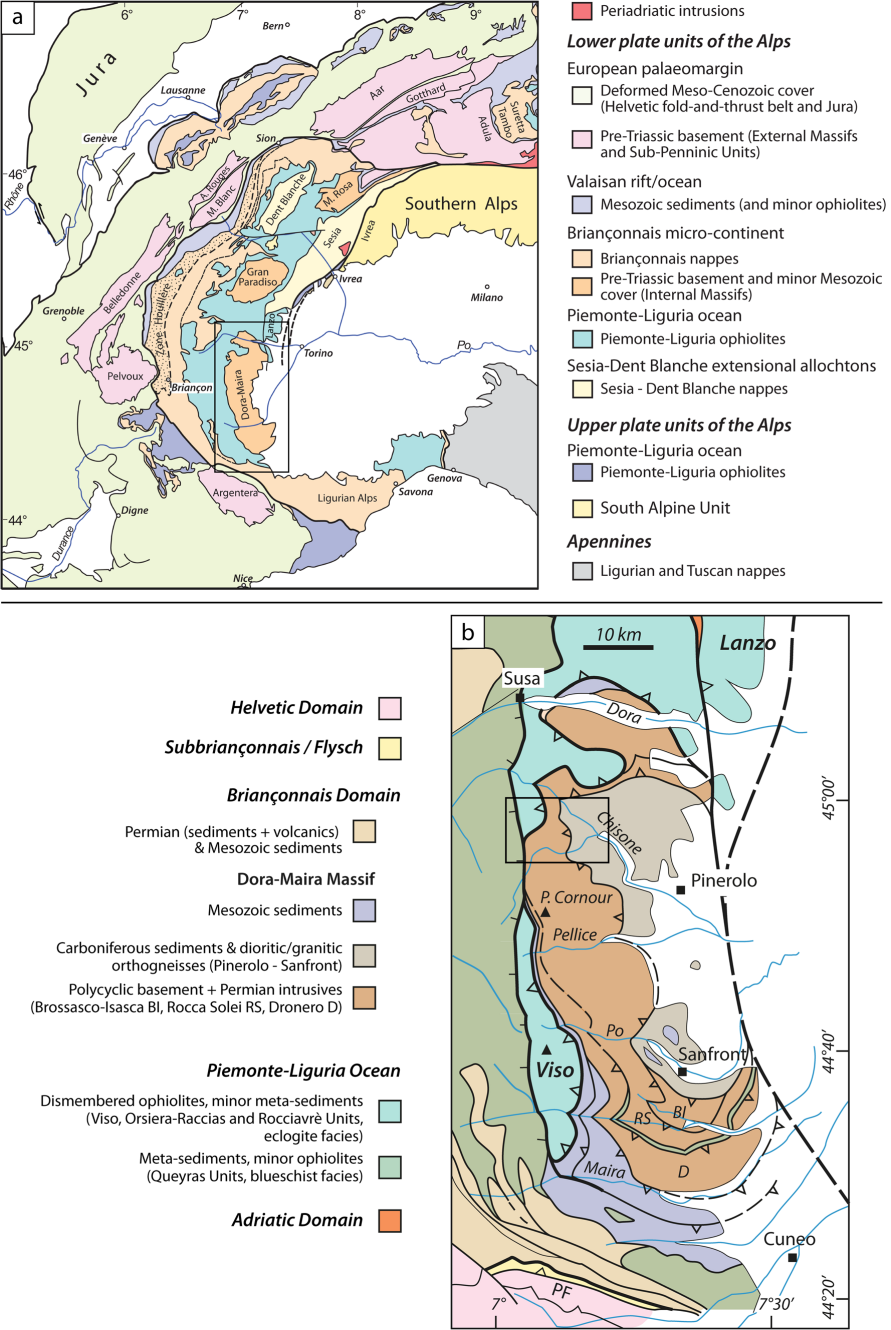
**a** Schematic structural map of the Western and Central Alps (modified after Schmid et al., 2004, 2017; Ballèvre et al., 2018) The black rectangle indicates the location of the tectonic map of **b**. **b** Tectonic map of the South-Western Alps (modified from Ballèvre et al., 2020). The black square indicates the location of the geological map of Fig. 2

The Dora-Maira Massif is located in the Western Alps (Fig. 1a) and extends for ~ 70 km from the Susa Valley in the north (Dora Riparia River) to the Maira Valley in the south (Fig. 1b). Geographically, it can be divided along the Pellice Valley into a northern sector, comprising the Susa, Chisone and Germanasca Valleys, and a southern sector, including the Po, Varaita and Maira Valleys. It mainly consists of a Palaeozoic basement (e.g. Vialon, 1966; Michard, 1967; Sandrone et al., 1993; Compagnoni & Rolfo, 2003) that was involved in the Mesozoic crustal thinning and reworked by the Alpine (Cenozoic) orogenesis (e.g. Schmid et al., 2004; Handy et al., 2010; Ballèvre et al., 2020; Michard et al., 2022). During the Mesozoic the (future) Dora-Maira Massif was part of the distal palaeomargin of the Briançonnais microcontinent, facing the Piemonte-Liguria Ocean.

The Dora-Maira Massif is overlain by the eclogite-facies ophiolites and their meta-sedimentary cover belonging to the Orsiera-Rocciavré Unit in the Chisone Valley (e.g. at the summit of the Punta Raccias) and Susa Valley, while in the Germanasca Valley it is directly overlain by the blueschist-facies meta-sediments belonging to the Queyras Unit (Fig. 1; Pognante, 1979; Cadoppi et al., 2002; Gasco et al., 2011; Ghignone et al., 2020).

The entire nappe pile is affected by a late, NS-trending, antiformal fold, which is attributed to the indentation of the Adriatic mantle (Schmid & Kissling, 2000; Schmid et al., 2017). As a consequence, the Dora-Maira Massif acquired a domal shape. Following erosion of the Alpine belt, and development of the foreland basin in front of the Apennine belt, almost half of the antiformal stack was covered by Pliocene to Recent deposits belonging to the Po Basin (Fig. 1). The studied transect along the Germanasca Valley therefore exposes the central and western part of the dome.

Within the entire Dora-Maira Massif, several tectonic units with different pre-Alpine and Alpine histories are now found stacked together (Fig. 1b). In the southern Dora-Maira Massif, burial of continental crust at mantle depth was demonstrated for the first time with the discovery of coesite (Chopin, 1984). Since then, several studies have contributed to establish the architecture of the tectonic stack in the southern Dora-Maira Massif where several *HP* units and one *UHP* unit differ for lithology and/or Alpine peak *P–T* conditions, and are separated by tectonic boundaries (e.g. Chopin et al., 1991; Avigad, 1992; Michard et al., 1993; Avigad et al., 2003; Compagnoni et al., 2012; Groppo et al., 2019). Further North, a pioneering study in the Pellice Valley has been performed by Wheeler (1991). However, the tectonic architecture of the northern Dora-Maira Massif remains poorly known, especially in the Germanasca and Chisone valleys. Geological maps of the area are ultimately based on the work of Mattirolo (1913, 1st edition; 1951, 2nd edition) and Vialon (1966). Further improvement of these works has been provided by Borghi et al. (1984) and Cadoppi et al. (2016). Meanwhile, recent studies in this area have been defining new tectonic units (Manzotti et al., 2022; Nosenzo et al., 2022). In line with these works, we aim to reconsider the tectonic stack of the northern Dora-Maira Massif and characterize the tectonic units therein.

## Methods

### Field work

Field work was carried out on the northern slope of the Germanasca Valley, and in the Bourcet and Garnier Valleys (all of which are tributaries of the Chisone Valley, Italy), over an area of about 140 km^2^. Field work aimed to identify the main lithologies, in order to distinguish the different units, and to define their boundaries as accurately as possible for such a densely forested area (the study area is located below the tree line). Lithological data have been reported on the CTR (Carta Tecnica Regionale) topographic map at the scale 1:10,000. GPS coordinates of all samples and sites of interest have been systematically recorded. In order to control field observations, a large number of thin sections (about 200) have been made.

**Fig. 2:**
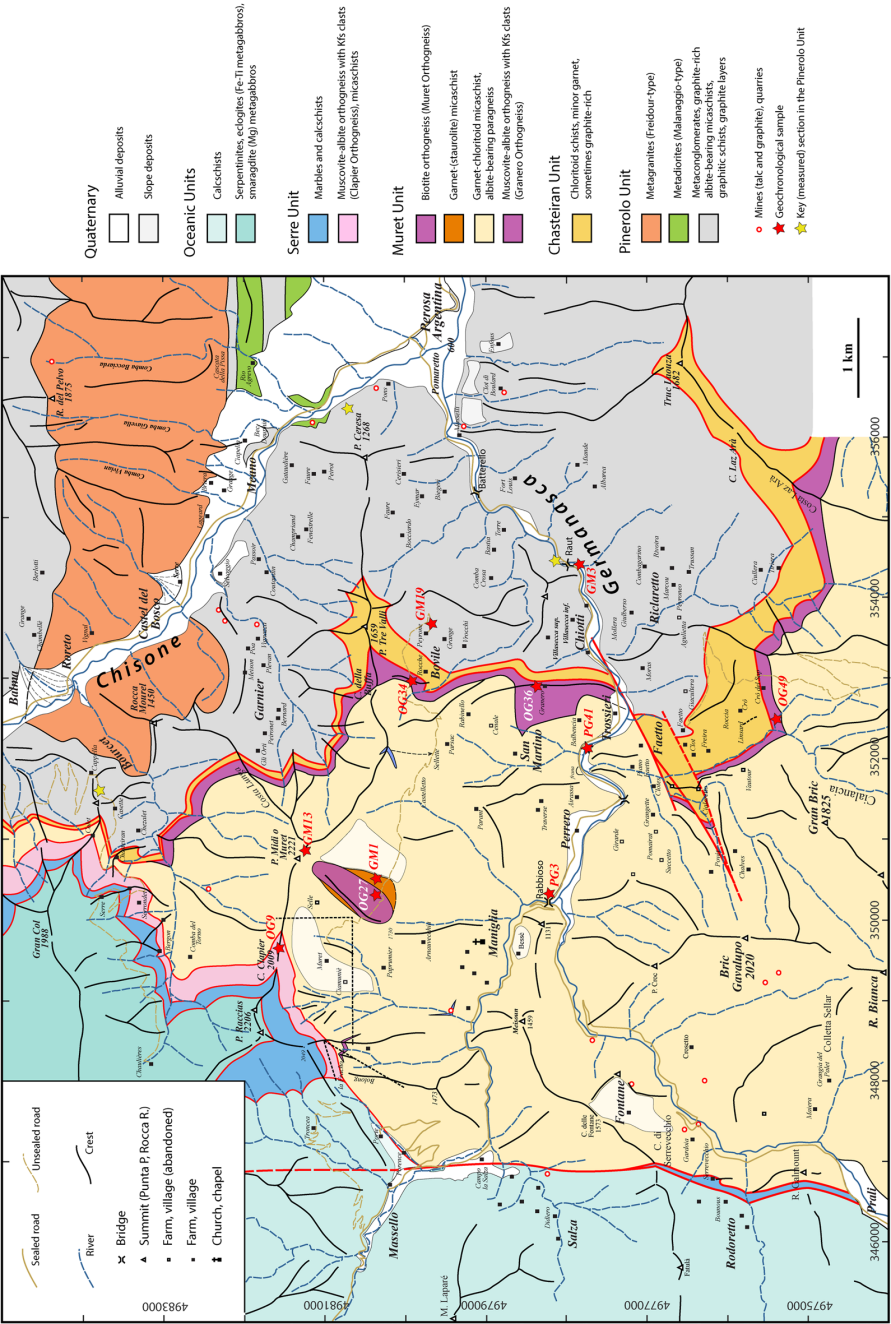
A simplified tectonic map of the northern Dora-Maira Massif in the Chisone and Germanasca valleys. Numbers associated with red stars refer to samples used for geochronology in this paper (i.e., OG34, OG36, OG49, OG9, PG3, PG41), in Nosenzo et al., 2022 (i.e. GM1, GM13, OG27) and in Manzotti et al. 2016 (i.e. GM3, GM19)

In the study area, like in most Alpine valleys, there is a striking difference between the slopes exposed to the North and those exposed to the South. While the former are covered by a dense forest, the latter are frequently subjected to landslides, which can have very large proportions (the so-called deep-seated gravitational slope deformation DSGSD; e.g. Forno et al., 2022). In the study area, glacial erosion and coeval glacial deposits (i.e. tills) are limited to the Cialancia valley (Fig. 2). By contrast, landslides are a characteristic feature of the southern slopes along the ridge between Punta Raccias and Punta Tre Valli, unfortunately obscuring the structural relationships in the pre-Alpine basement (Fig. 2).

**Fig. 3:**
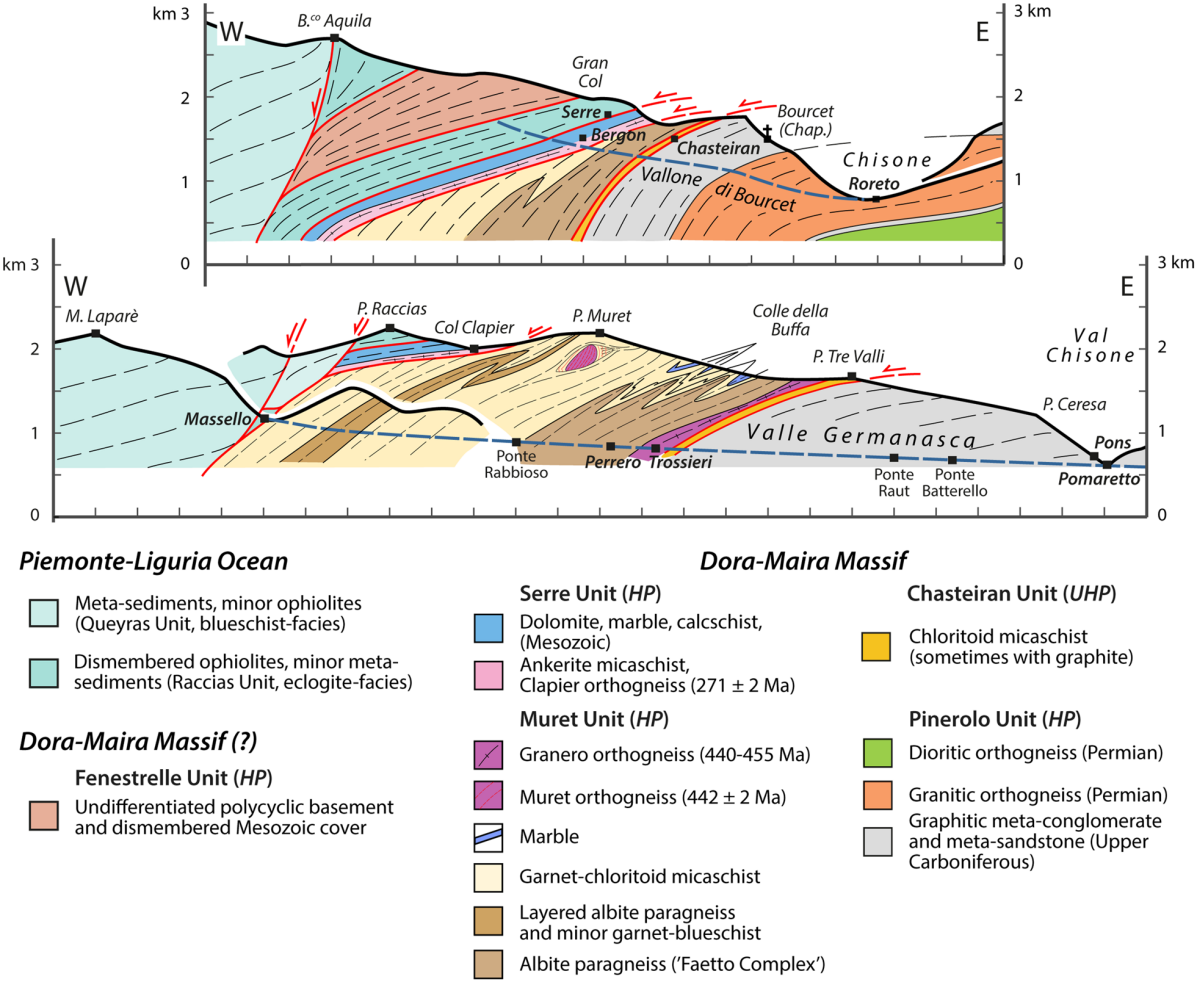
Two schematic cross-sections of the northern Dora-Maira Massif (modified from Manzotti et al., 2022). Tectonic boundaries are shown in red. The trend of the main, regional, foliation S_2_ is indicated by thin, dashed, lines. Kilometre-scale folding a previous schistosity S_1_ are indicative only, being recognized with certainty only in a few cases. A pre-Alpine foliation (thin red dashed lines) has been identified in a volume poorly deformed during the Alpine orogeny and located below the Punta Muret

The data are summarized on a simplified tectonic map (Fig. 2) compiled on the topographic map at 1:25,000 scale. In addition, we constructed two simplified cross-sections aimed at displaying the geometrical relations between the different units (Fig. 3).

### Whole-rock geochemistry

The major element composition of five orthogneiss samples (Table 1) was measured by X-ray fluorescence (XRF) at the PetroTectonic analytical facility at Stockholm University. A specimen of ~ 0.5 dm^3^ in volume was extracted from the hand sample excluding veins and weathered surfaces and subsequently crushed, mixed with di-lithium tetraborate in proportion 2:5 and fused. A list of the obtained whole-rock compositions is given in Additional file 1: Table S1.

**Table 1:** Summary of samples used for whole-rock geochemistry and geochronology

Lithology	Sample name	Locality	Lat/long coordinates	XRF	U/Pb geochronology
Granero orthogneiss	OG34	Climbing school of Bovile	44° 57′ 27.1′′ N–7° 8′ 11.3′′E	X	X
	OG36	Granero	44°56′33.4′′ N–7°8′8.8′′ E	X	X
	OG49	Rio di Faetto	44°54′58.9′′ N–7°7′54.0′′ E	X	X
Clapier orthogneiss	OG7	La Fracho	44°57′53.3′′ N–7°4′36.4′′ E	X	
	OG9	Col Clapier	44°58′18.4′′ N–7°5′32.0′′ E	X	X
Paragneiss	PG3	Ponte Rabbioso	44°56′29.0′′ N–7°6′7.1′′ E		X
	PG41	Trossieri	44°56′16.0′′ N–7°7′35.5′′ E		X

### Zircon U-Pb geochronology and trace element geochemistry

Zircon crystals were separated from four orthogneiss and two albite-rich paragneiss samples (Table 1). About 1.7 dm^3^ of rock material was crushed and milled with a Resch PM400 tungsten steel mortar at Stockholm University. Zircon grains were separated from the light mineral fraction with a Wilfley Table. Zircon crystals were handpicked and mounted on a one-inch epoxy resin puck, subsequently polished to expose the equatorial section of the crystals. Particular attention was dedicated to the paragneiss samples in selecting grains with different size, shape and colour in order to limit the handpicking biases in a detrital zircon population (Sláma & Košler, 2012). Cathodoluminescence (CL) imaging was carried out with a XL30ESEM-FEG equipped with a Centaurus detector (15 kV accelerating voltage) at the Swedish Museum of Natural History (NRM). U–Pb geochronology and trace element geochemistry of zircon was conducted by in situ LA-ICP-MS with an ESI NWR193UC Excimer laser coupled to an Agilent quadrupole 7700 × ICP-MS at the GeOHeLiS analytical platform (University of Rennes, France). The trace element contents of zircon were analysed only for the orthogneiss samples. To maximize analytical precision and accuracy, zircon ages and trace element contents were measured on separate ablation spots on the same crystal. This was possible because zircon in the studied samples generally displays a simple internal composition with relatively large (~ 100 μm) cores only locally surrounded by very thin rims (~ 5 μm thick). The rims were not analysed because they are too thin. The ages were measured during a first analytical session and the trace element contents were measured during a second session. The trace element content was analysed preferentially in the crystals which yield the most concordant ages. Details on the instrument working conditions are reported in Additional file 1: Appendix S1 and Appendix S2 for zircon ages and trace element contents, respectively. A spot size of 25 μm was used for both age and trace element analyses. Measurements of the unknown was bracketed with repeated measurements of primary reference materials. The latter was the GJ1 zircon (Jackson et al., 2004) for U–Pb dating and the NIST-612 glass for trace element contents. The Plešovice standard zircon (Sláma et al., 2008) was used as a secondary reference material to monitor precision and accuracy of the U–Pb data and yielded a concordia age of 336.9 ± 1.7 Ma (95% c.i.; MSWD (concordance + equivalence) = 1.0; n = 16) and 336.8 ± 1.1 Ma (MSWD = 0.74; n = 48) during the two main analytical sessions conducted. The 91,500 standard zircon was used as a secondary reference material for trace element (Wiedenbeck et al., 1995; 2004). Raw data were processed with the software Iolite v4 (Paton et al., 2010, 2011). The reproducibility of the secondary reference material Plešovice has been propagated by quadratic addition for the individual analyses according to Horstwood et al. (2016). Concordia ages and diagrams were generated with IsoplotR (Vermeesch, 2018). Concordia ages are given with 95% confidence level. Mean squared weighted deviations (MSWD) are given for concordance plus equivalence. Rare earth elements (REE) patterns were normalized to chondrite (McDonough and Sun, 1995). Detrital ages were plotted in Kernel density diagrams generated with the program DensityPlotter (Vermeesch, 2012) using an adaptive bandwidth. The detrital datasets for discordance analyses were filtered using a 10% threshold of concordance. This approach has been adopted in a previous detrital zircon investigation conducted in the Pinerolo Unit (Manzotti et al., 2016) and therefore allows a reliable comparison for all datasets. In the text and figures, individual dates are given as ^206^Pb/^238^U dates if they are < 1000 Ma and as ^207^Pb/^206^Pb dates if they are > 1000 Ma. The relative error is given as 2σ. The distinction between magmatic or metamorphic sources is based on the Th/U ratios (cf. Hoskin and Schaltegger, 2003; Teipel et al., 2004) and on the internal zoning displayed by the crystals. The maximum age of deposition from detrital zircon U–Pb data was determined calculating the youngest age population on the basis of the weighted mean of at least three analyses that agree at 2σ (Dickinson and Gehrels, 2009). The complete dataset of zircon U–Pb ages and trace element is given in Additional file 2: Table S2.

## Lithologies of the main units

Our mapping in the Germanasca and Chisone valleys allows us to distinguish several units (Figs. 2, 3), whose lithological contents are briefly described below. In agreement with previous works, we identify a monocyclic unit at the base (Pinerolo Unit), which is overlain by a polycyclic unit (Muret Unit: Nosenzo et al., 2022 and 2023). However, a close examination of the boundary between these two major units led to the discovery of a new, UHP, unit (the Chasteiran Unit: Manzotti et al., 2022) as well as a redefinition of their contents and geometries, as detailed below. The units are described from bottom to top.

### Pinerolo unit

#### Meta-sediments from the Pinerolo Unit

**Fig. 4:**
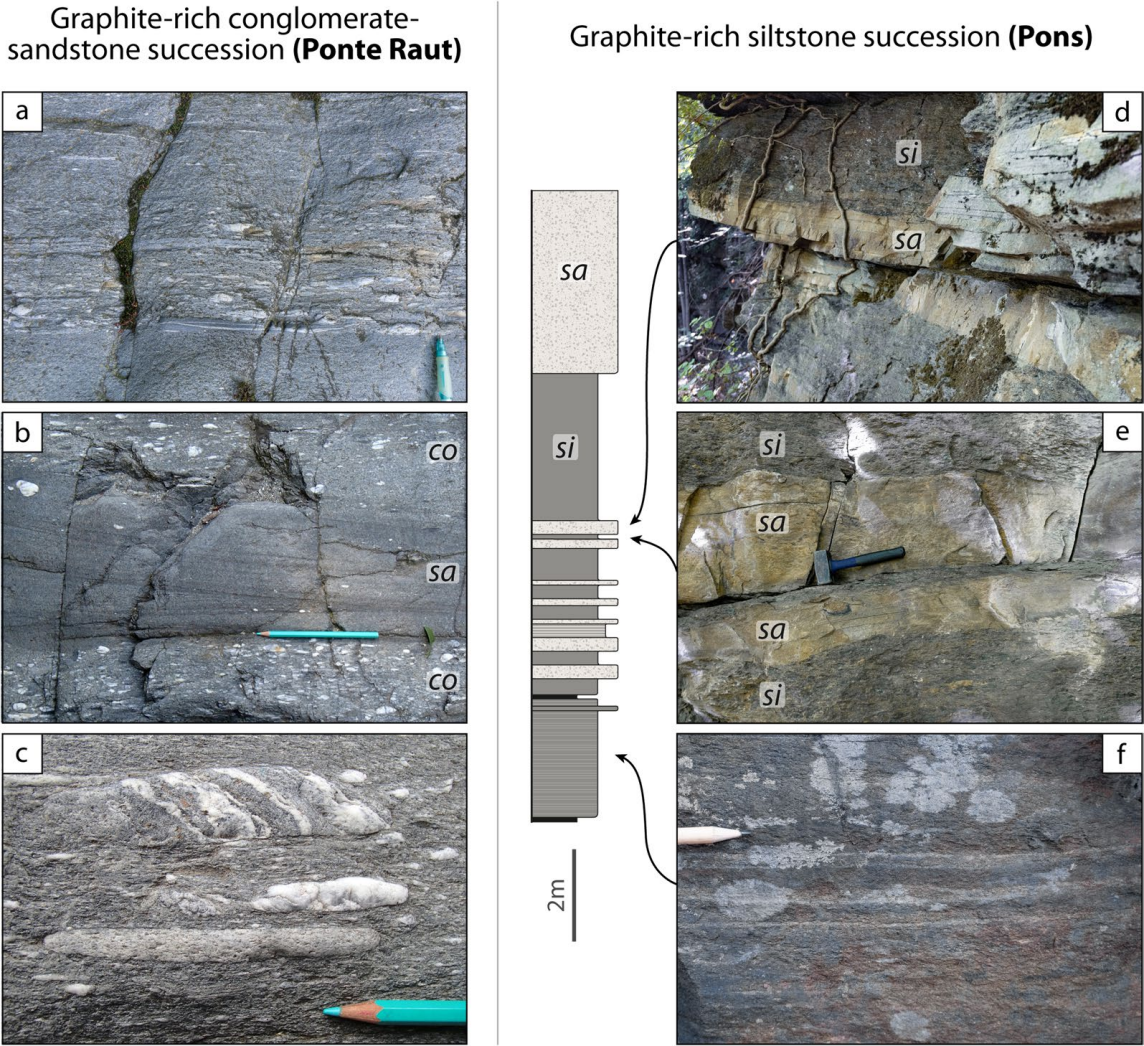
Field aspect of the meta-sediments within the Pinerolo Unit: graphite-rich conglomerate-sandstone succession (**a–c,** 44° 56′ 24.8′′ N–7°9′ 18.2′′ E) and graphite-rich siltstone succession (**d–f,** 44° 58′ 11.9′′ N–7° 10′ 28.7′′ E) (si = meta-siltstones, sa = meta-sandstones, co = meta-conglomerates). In the stratigraphic log the reported thickness of the layers is the one measured on the outcrop and, thus, represents the thickness after the Alpine strain. **a** Graded meta-conglomerate layer with an upward polarity. **b** Interbedded meta-conglomerates and meta-sandstones in decimetre-thick layers. Meta-sandstones preserve a cross-bedding lamination. **c** Centimetre-sized pebbles of garnet-bearing micaschist, felsic gneiss and quartz in polygenic meta-conglomerates. **d**, **e** Graphite-poor meta-sandstone layers interbedded with graphite-rich meta-siltstones. **f** Meta-siltstones with a millimetre-thick lamination defined by the occurrence of albite-rich laminae

**Fig. 5:**
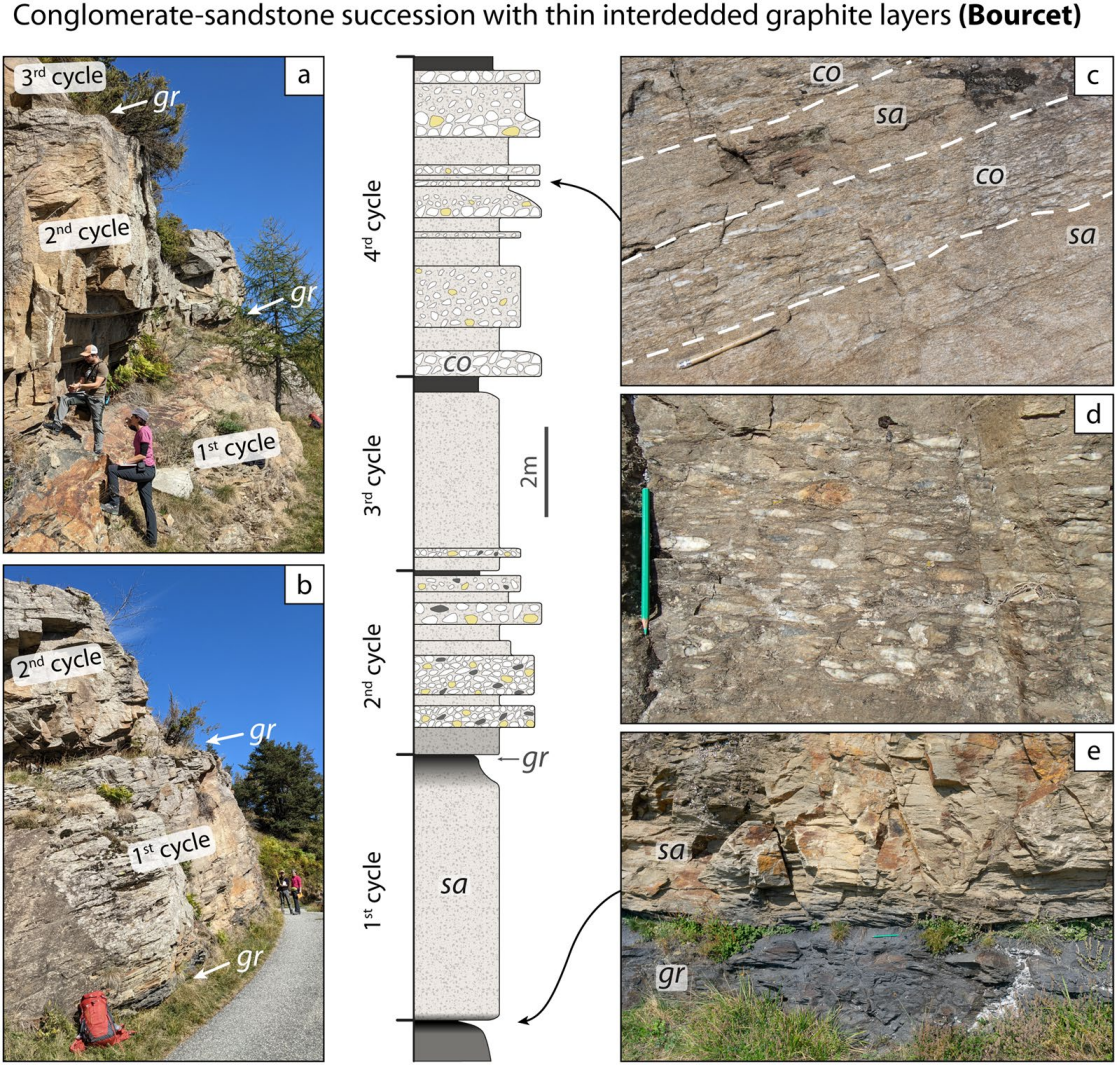
Field aspect of the meta-sediments within the Pinerolo Unit: conglomerate-sandstone succession with interbedded graphite layers (gr = graphite layer). In the stratigraphic log the reported thickness of the layers is the one measured on the outcrop and, thus, represents the thickness after the Alpine strain. White, yellow and grey ovals represent quartz-, feldspar- and graphite-rich pebbles, respectively. **a b** Outcrop aspect consisting of cliffs (a few metres thick) separated by ledges in correspondence of graphite layers, interpreted as resulting from a cyclic sedimentation. **c** Interbedded meta-conglomerates and meta-sandstones. **d** Polygenic meta-conglomerates with centimetre- to decimetre-sized pebbles of graphite-rich schist, interpreted as original clay chips, and centimetre-sized pebbles of quartz and felsic gneiss. **e** Graphite layer at the base of thick sandstone layer. **a d** Lat/long coordinates 44° 59′ 31.9′′ N–7° 07′ 05.3′′ E. **b c e** Lat/long coordinates 44° 59′ 32.3′′ N–7° 07′ 06.8′′ E

The lowermost unit in the studied area is the Pinerolo Unit, which is characterized by graphite-bearing meta-sediments that were mined for graphite in the late 1800s and early- to mid-1900s (e.g. Novarese, 1898, 1905; Jannin and Magrì, 2017; Bounous, 2018). Despite the strong Alpine deformation and metamorphism, it is possible to identify some characteristics of the sedimentary protoliths, namely the cm- to m-scale bedding, the mm- to cm-scale lamination, the differences in grain size and sorting, and the differences in colour, reflecting the amount of organic matter. This allows to infer some of the depositional environments. The Pinerolo Unit is known for its meta-conglomerates, best seen along the river Germanasca in the section between Ponte Batterello and Ponte Raut (Fig. 2) (e.g. Vialon, 1966; Mertz and Siddans, 1985; Manzotti et al., 2016; Petroccia and Iaccarino, 2022). According to our field work, conglomeratic layers are not homogeneously distributed throughout the unit and some areas have no meta-conglomerates at all. It appears that three kinds of sedimentary successions can be identified. They differ for bedding thickness, granulometry and organic matter content of the protoliths. To document this, we measured two representative stratigraphic successions observed in two type-localities, namely Bourcet and Pons (Figs. 4, 5). Due to poor outcrop conditions, we have not been able to measure a representative succession along the Germanasca river (Ponte Raut). Because of the Alpine deformation, we are well aware that our measurements do not represent the original bed thickness. However, identifying the main sedimentary protoliths, their succession and their relative proportions still gives a first-order information on the past sedimentary environments.

*Graphite-rich conglomerate-sandstone successions (Ponte Raut)*

These successions constitute the classic outcrops along the Germanasca Valley between Ponte Batterello and Ponte Raut. All lithologies are grey in colour, due to abundant graphite finely dispersed in the sand-size and silt-size fraction that constitutes the finer-grained matrix of the meta-conglomerates and meta-sandstones (e.g. at Ponte Raut locality, 44°56′24.9′′ N–07° 09′18.0′′ E; Fig. 4a–c). The clasts within the meta-conglomerates range from gravel-size to pebble-size (up to 15 cm long, after the Alpine deformation) and consist of (in decreasing order of abundance) quartz, felsic gneiss and graphite-rich fine-grained schist with an internal foliation parallel to the main Alpine fabric in the matrix. Rare examples of pebbles of garnet-bearing micaschist locally occur, and they display an internal foliation discordant with the main Alpine fabric in the matrix (Fig. 4c). Meta-conglomerates display variable proportions of clasts with respect to the matrix, and locally preserve graded bedding as well as cross bedding (Fig. 4a, b). The matrix consists of quartz, albite and phyllosilicates. Meta-sandstone layers locally display a planar or cross-bedding lamination defined by the alternation of laminae with different granulometry ranging from silt-size to sand-size and locally up to gravel-size (Fig. 4b). Meta-conglomerate and meta-sandstone layers may be proved to be laterally discontinuous, provided outcrop conditions are good enough.

*Graphite-rich siltstone successions (Pons)*

A different succession, dominated by very fine-grained meta-sediments, can be observed, for example, close to the village of Pons (44°58′11.9′′ N–07°10′28.7′′ E; Fig. 4d–f). It is characterized by meta-siltstones with interbedded meta-sandstones forming layers from a centimetre to a few cm-thick (Fig. 4d, e). Meta-conglomerates are absent. Coal layers, up to 20 cm thick, transformed into graphite, occur episodically within the sequence, although they are not found at regular intervals. Meta-siltstones are grey in colour due to the abundance of graphite finely dispersed in the matrix and display a well-defined foliation. Locally meta-siltstones display a millimetre-thick lamination defined by the local abundance of albite (Fig. 4f). Meta-sandstones are generally poorer in graphite and display a poorly developed foliation, due to the lower amount of mica compared to quartz and albite. Locally meta-sandstones have a planar lamination defined by the alternation of millimetre-thick layers with different albite and graphite content.

*Conglomerate-sandstone successions with thin interbedded graphite layers (Bourcet)*

An easily accessible locality for this type of succession is found in the Bourcet Valley, along the road to Chasteiran, east of the former Bourcet Chapel (44°59′32.3′′ N–07°07′6.8′′ E). Another nice example of this type, less easily accessible, has been found to the south of Punta Tre Valli (44°57′40.1′′ N–07° 09′0.5′′ E). The outcrop aspect is very characteristic with steep cliffs, a few metres high, separated by narrow ledges due to the erosion of very fine-grained, black, graphite-rich schists (up to 40 cm thick), interpreted as former organic-rich mudstones and/or coal layers (Fig. 5). Detailed measurements on the Bourcet outcrop shows that 3–5 m thick cliffs consist either of a homogeneous meta-sandstone layer or of a decimetre- to metre-thick alternation of meta-conglomerates and meta-sandstones (Fig. 5c). The relative proportion of meta-conglomerates vs. meta-sandstones may change from one cliff to the other. Graphite in meta-conglomerates and meta-sandstones is generally low in modal amount, conferring them a beige to pale grey colour. The clasts within the meta-conglomerates generally range from gravel to pebble size (up to a few centimetres large) and consist, in decreasing order of abundance, of quartz, felsic gneiss and graphite-rich fine-grained schist (Fig. 5d).

*Age of the sediments*

Since Novarese (1895a, 1895b, 1898), the protoliths of the Pinerolo Unit meta-sediments are considered Carboniferous in age, based on their characteristic lithology. The maximum age of deposition has been constrained by detrital zircon geochronology, which yielded a youngest and most abundant population at 340–330 Ma, consisting predominantly of crystals with a magmatic signature (i.e. they do not yield metamorphic ages; Manzotti et al., 2016).

#### Intrusive rocks in the Pinerolo Unit

Large volumes of meta-intrusives of felsic and intermediate composition occur within the Pinerolo Unit. A large orthogneiss body, the Freidour orthogneiss, extends from the Sangone Valley (Monte Freidour), east of our study area, to the Chisone Valley (Rocca Mourel). It consists of an augen gneiss with variable amount and size of alkali feldspar porphyroclasts (up to a few centimetres in size) in a matrix of quartz, albite, white mica, biotite and epidote (Borghi et al., 2016; Cadoppi, 1990). A few decimetre-thick fine-grained quartz-feldspathic layers occur within the meta-sediments and are parallelized to the main Alpine foliation, possibly representing former aplitic dykes or sills genetically connected with the Freidour orthogneiss (e.g. 44°57′30.70′′ N, 7°09′29.12′′ E).

Hectometre- to kilometre-scale bodies of meta-diorite and meta-quartzdiorite crop out in the Chisone Valley (Sandrone et al., 1988; e.g. at Brandoneugna where it is actively quarried, 44° 58′ 11.6′′ N, 7° 10′ 38.3′′ E). These bodies are generally referred to as the Malanaggio diorite (Bussy and Cadoppi, 1996), the type locality being in the lower Chisone Valley, out of our study area. It is worth noting that the different dioritic bodies are not necessarily connected. The Malanaggio diorite displays domains rich in albite and epidote (sites of the former magmatic plagioclase) and domains of chlorite, epidote and actinolite (sites of the former magmatic hornblende), generally stretched along the main Alpine foliation. Lenticular melanocratic enclaves are locally abundant (Borghi et al., 2016).

The magmatic protoliths of the Freidour orthogneiss (268–273 Ma: Bussy and Cadoppi, 1996) and the Malanaggio meta-diorite (288 ± 2 and 290 ± 2 Ma: Bussy and Cadoppi, 1996) are Permian in age.

### Chasteiran Unit

**Fig. 6:**
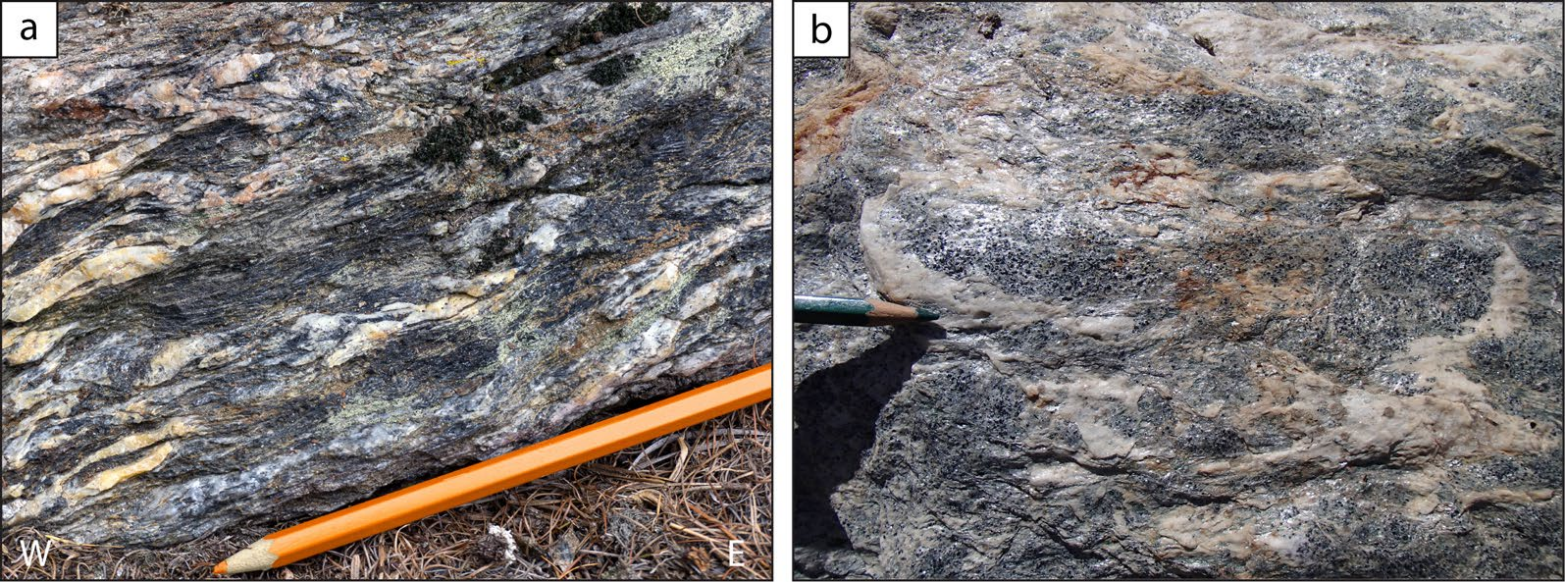
**a b** Field aspect of chloritoid micaschist of the Chasteiran Unit (44° 57′ 43.2′′ N–7° 08′ 51.4′′ E and 44° 55′ 18.39′′ N–7° 10′ 55.37′′ E). In the section perpendicular to the foliation (**a**), numerous quartz veins are parallel to elongate, black, lenses essentially consisting of chlorioid and muscovite (**a**). The quartz veins are microfolded, with the S_1_ schistosity being sometimes preserved at the macroscopic scale. The main, regional foliation is, hower, a composite S_1–2_ foliation. Chloritoid flakes are best seen in a surface parallel to the foliation planes (**b**). Note in both images the scarcity of mm-sized garnet crystals

The Chasteiran Unit directly overlies the Pinerolo Unit (Manzotti et al., 2022). It is only 10–50 m thick but it is laterally continuous. According to our observations, it extends from Villaretto (Chisone Valley) to the crest of Truc Laouza (Germanasca Valley, Fig. 2). The Chasteiran Unit consists of chloritoid micaschists with a distinctive field aspect characterized by the occurrence of thin and discontinuous layers/lenses of graphite (generally less than a millimetre and up to a few millimetres thick). Thus, differently from the meta-sediments of the Pinerolo Unit, graphite is mainly concentrated in microscale domains, although a minor quantity is also dispersed in the rock. Micaschists display a mylonitic foliation defined by graphite layers, lenticular mica- and chloritoid-rich layers (up to a centimetre thick) and quartz-rich lenses (Fig. 6). Garnet is rare and displays coesite inclusions (full details are reported in Manzotti et al., 2022).

### Muret Unit

The Muret Unit is well-exposed in the Germanasca Valley (Nosenzo et al., 2022) where it reaches a thickness of 2 km and becomes very thin (only a few tens of metres thick) in the Bourcet Valley. The Muret Unit comprises several lithologies ascribed to a pre-Carboniferous basement.

#### The Granero Orthogneiss

A thin tabular layer of orthogneiss, hereafter named Granero Orthogneiss, directly overlies the micaschists of the Chasteiran Unit (Fig. 2). It can be followed for several kilometres, discontinuously outcropping in the forest. In accordance with previous maps (Novarese, 1895a; Vialon, 1966; Borghi and Sandrone, 1990), we have recognized the orthogneiss on the right side of the Bourcet Valley, at Costa Lunga (i.e. the ridge between Punta Muret and Rocca Mourel) and then along the northern slope of the Germanasca Valley, nicely cropping out at Colle della Buffa, at Granero, and east of Trossieri (Fig. 2). The same orthogneiss is also present along the southern slope of the Germanasca Valley, where we discovered new outcrops in the tributary Faetto Valley, making the link with the outcrops already reported along the Costa Laz Arà. The Granero orthogneiss has not been recognized either in the Chisone Valley or on the left side of the Bourcet Valley. As a whole, the orthogneiss body is about 8 km long, and its thickness varies from 50 to 200 m (being the thickest at Granero).

**Fig. 7:**
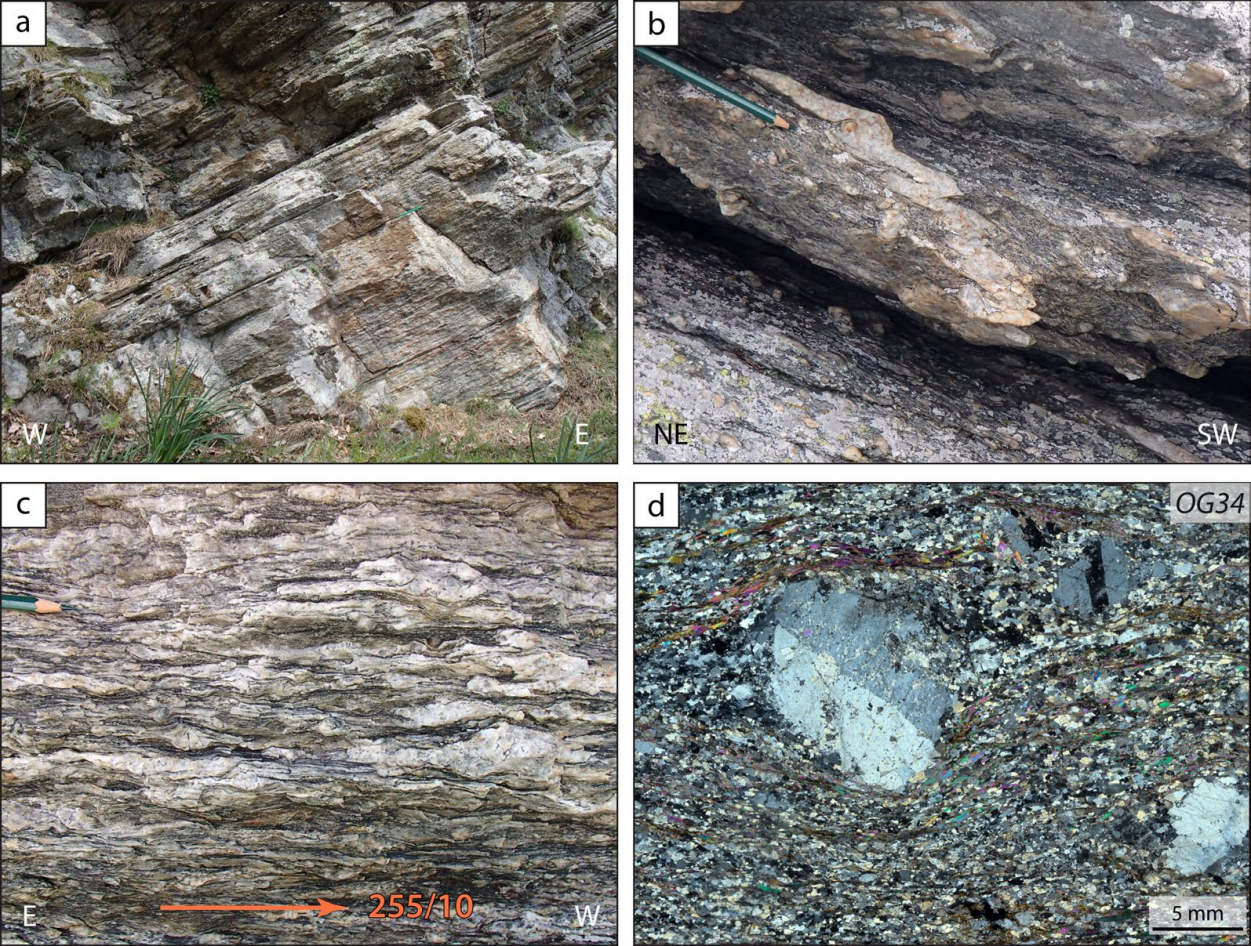
Field aspect and petrographic characterization of the Granero Orthogneiss. **a** An outcrop displaying the characteristic, regular and tabular, foliation in the orthogneiss, indicating strong flattening during ductile deformation (44° 55′ 26.31′′ N, 7° 06′ 49.9′′ E). **b** A folded quartzo-feldspathic layer in the augen orthogneiss, interpreted as a former aplitic dyke. Note that an early foliation, parallel to the boundaries of the meta-aplitic dyke, is observed in the orthogneiss. The asymmetry of the folds is consistent with a top to the W sense of shear (44° 54′ 51.77′′ N, 7° 10′ 02.46′′ E). **c** A section perpendicular to the foliation and parallel to the stretching lineation evidences the strong flattening of the orthogneiss body. Note that narrow shear bands (C’) indicate top to W sense of shear (44° 54′ 55.8′′ N, 7° 10′ 05.4′′ E). **d** Cross-polarised light photomicrograph of the Granero orthogneiss (sample OG34, 44° 57′ 27.1′′ N–7° 08′ 11.3′′ E) showing a millimetre-size alkali feldspar with a simple twin and exsolution lamellae (perthite)

The Granero Orthogneiss consists of augen gneisses with a variable amount of alkali feldspar porphyroclasts (with different sizes up to 3 cm; Fig. 7), locally interlayered with minor fine-grained felsic leucocratic gneisses lacking porphyroclasts. Within the feldspar porphyroclasts, it is locally possible to distinguish an inner core with a bluish colour, likely the relict of the magmatic crystal, surrounded by a thick recrystallized corona with a whitish colour. Other than alkali felspar and zircon, no magmatic relicts have been identified. The relative amount of feldspar, quartz and muscovite in the matrix varies in different decimetre- to metre-scale layers, suggesting that magmatic rocks with different compositions were involved. The variability in size and abundance of the alkali feldspar porphyroclasts may also be due to strain localization. The mylonitic foliation (Fig. 7a) is defined by the shape fabric of muscovite. Flattened enclaves have not been recognized. Folded fine-grained leucocratic layers are interpreted as former aplitic dikes (Fig. 7b).

#### Lithologies in the low-strain domain within the Muret Unit

South of the Punta Muret a kilometre-scale body occurs where the Alpine deformation is absent or very weak, allowing recognition of the pre-Alpine protoliths (Nosenzo et al., 2022, 2023). Since a complete description of these lithologies has already been made, we will only recall some of the most important results.

The Muret Orthogneiss (~ 2 km long and ~ 300 m thick) is an augen gneiss displaying dispersed alkali felspar porphyroclasts, interpreted as magmatic relicts, in a foliation defined by quartz, plagioclase, Ti–rich biotite, low-Si muscovite and garnet. Abundant, strongly flattened, enclaves are found in the orthogneiss. The amphibolite-facies foliation, pre-Alpine in age, is parallel to the margins of the orthogneiss body and to the pre-Alpine foliation in garnet-staurolite micaschists found along its contact. These micaschists contain large (up to 2 cm) garnet porphyroblasts and greenish, elongated rhomboidal prism (up to 1.5 cm long), essentially made of chloritoid and mica, interpreted as pseudomorphs after pre-Alpine staurolite. In the micaschists, numerous quartz-tourmaline veins are observed. The protolith of the Muret Orthogneiss has been dated at 442 ± 2 Ma (Nosenzo et al., 2022), and is supposed to have intruded the protolith of the micaschists. Both were deformed during an amphibolite-facies episode (6–7 kbar, 650 °C), dated at 324 ± 6 Ma (Nosenzo et al., 2022).

The boundaries of the low-strain domain cannot be precisely defined in the field, due to poor outcrop conditions. The Muret Orthogneiss defines a crest on the southern slope of the Punta Muret, and its contact with the garnet-(staurolite) schists may be observed on both side of the orthogneiss body (Nosenzo et al., 2023, Fig. 1b). However, the transition toward the high-strain domain is obscured by large-scale landslides.

#### Lithologies in the high-strain domain within the Muret Unit

The bulk of the Muret Unit is essentially made of meta-sediments that have been pervasively deformed during the Alpine orogenesis. Two main type of meta-sediments have been distinguished in the field, namely garnet-chloritoid micaschists and paragneisses. Other lithologies (marbles and meta-basites) occur in very limited amounts in the studied area.

*Garnet-chloritoid micaschists*

The garnet-chloritoid micaschists, largely outcropping in the Germanasca, Prali, Massello, and Chisone Valleys, display millimetre-thick lenticular quartz-rich layers alternating with mica- and chloritoid-rich layers oriented parallel to the main Alpine foliation, locally microfolded. Graphite is generally scarce except in a few occurrences where it confers a grey colour to the rock (e.g. in the garnet-chloritoid micaschists at the summit of the Punta Muret, Nosenzo et al., 2022). In these micaschists, graphite is finely dispersed within the rock and does not concentrate in layers. Most garnet-chloritoid micaschists generally have a high modal amount of garnet. Two types of garnet can be found in the micaschists. A first group forms rather large grains (1–3 mm), whereas a second group forms minute grains (smaller than about 0.3 mm) barely visible with the naked eye but identifiable with the hand lens. In the Dora-Maira Massif, these two types have been interpreted as two generations of garnet (Sandrone and Borghi, 1992; Borghi et al., 1996), a pre-Alpine (larger grains) and an Alpine one (smaller grains). A detailed investigation of polycyclic garnet (Nosenzo et al., 2023) has shown that a single garnet type can also occur, and it consists of rather large grains (1 mm to 1 cm) comprising a pre-Alpine core and an Alpine rim overgrowth. The garnet-chloritoid micaschists are therefore indisputable polycyclic rocks. Micaschists with centimetric garnet porphyroblasts, which may have similar protolith to that of garnet-staurolite micaschist of the low-strain domain, are sporadically found in the high-strain domain (e.g. along the Costa Lunga ridge). However, their occurrence is very limited and pseudomorphs after staurolite are not preserved, possibly due to the intense Alpine deformation.

Decimetre- to metre-thick quartzite layers, poor in mica and chloritoid, are locally intercalated within the garnet-chloritoid micaschists and may derive from sandstones. Rare felsic leucocratic layers (decimetre-thick), interpreted as former aplitic dykes or sills, occur sporadically within the garnet-chloritoid micaschists and are oriented parallel to the main Alpine foliation.

*Paragneisses*

**Fig. 8:**
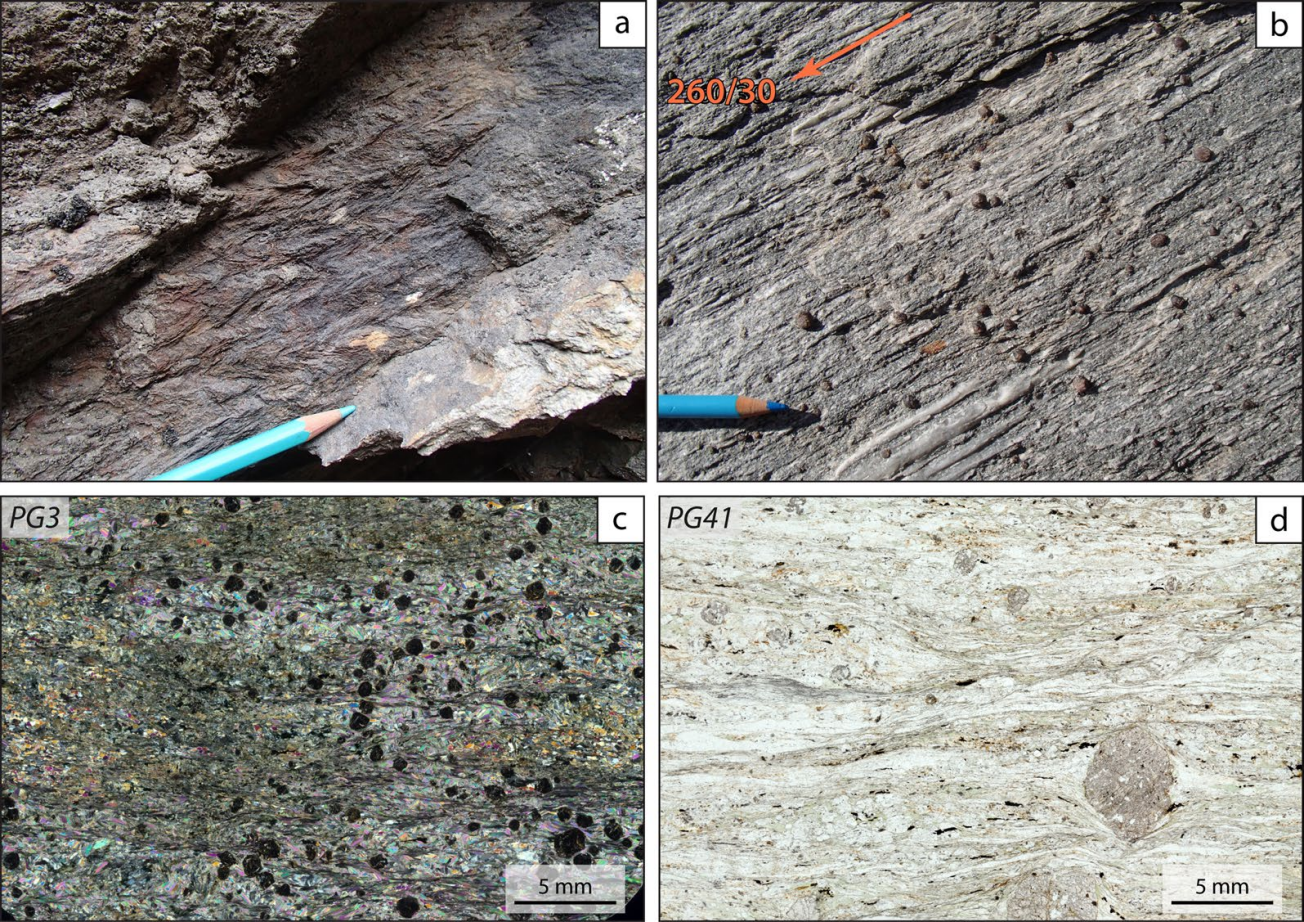
Field and petrographic aspects of the paragneiss exposed in the Germanasca valley, in the area of the Ponte Rabbioso (**a, c**; 44° 56′ 29.0′′ N, 7° 06′ 07.1′′ E) and close to Trossieri (**b, d**; 44° 56′ 16.0′′ N, 7° 07′ 35.5′′ E). The paragneiss cropping out close to the Ponte Rabbioso displays a pervasive crenulation (**a**) and numerous submillimetre-sized garnet. Close to Trossieri, the paragneiss are characterised by a well-defined stretching lineation (**b**) and by a few millimetre-sized garnet (**d**)

Paragneisses reach their maximum thickness (more than 1 km) along the Germanasca River (Fig. 2). The best outcrops are found above the Granero Orthogneiss, from Trossieri to Ponte Rabbioso (Fig. 8). They become very thin (10–15 m thick) towards the north, in the Bourcet Valley. Paragneisses are generally fine-grained and homogeneous at the sample scale, although they can display a millimetre- to decimetre-thick layering. They contain albite, quartz, white mica, chlorite, garnet and minor graphite, epidote, titanite, biotite in slightly different proportions in the different millimetre- to decimetre-thick layers. Euhedral to subhedral garnet is generally low in modal amount and very small in size (< 1 mm), but can be locally more abundant (Fig. 8b) and reach up to 8 mm in albite-poor layers (Fig. 8d). Because chloritoid is essentially absent but phyllosilicates are present is moderate amounts, we interpret the paragneisses as being derived from greywackes. Highly stretched millimetre- to centimetre-thick quarzitic layers and veins are locally intercalated within the paragneisses and are oriented parallel to the main Alpine foliation. Minor leucocratic felsic gneisses up to 10 cm thick, very rich in quartz, are sporadically interlayered within the paragneisses and oriented parallel to the main Alpine foliation.

*Albite- and epidote-bearing paragneisses associated with garnet blueschists*

The Muret Unit also comprises a few large volumes (from metre-scale to hectometre-scale) of layered paragneisses. They display a millimetre- to metre-thick layering defined by the different proportions of quartz, albite, garnet, white mica, biotite, chlorite, epidote and graphite. Rare veins and nodules of quartz or quartz + tourmaline, up to 10 cm large, are stretched parallel to the main Alpine foliation. Typically, the albite- and epidote-bearing paragneisses contain boudinaged mafic layers ranging in size from a few centimetres to several metres. A thick but discontinuous (~ 100 m thick and ~ 2 km long) mafic body crops out in the Massello Valley. Such mafic rocks consist of retrogressed garnet-bearing blueschists with a millimetre- to metre-thick layering defined by the different proportions of blue and green amphibole, chlorite, albite, epidote and rutile. Garnet locally forms porphyroblasts up to 5 mm in size in a generally fine-grained matrix (< 1 mm).

*Marbles*

Discontinuous layers of marbles with a whitish, bluish or greyish colour are intercalated within the garnet-chloritoid micaschists. They are essentially made of calcite, although some layers may also contain minor amounts of white mica, colourless pyroxene (diopside) and amphibole (tremolite), as previously reported (Cadoppi, 1990; Cadoppi et al., 1996). They crop out extensively in the Prali Valley (e.g. at Rocca Bianca) where they are locally associated with talc mineralization (Borghi et al., 2016; Cadoppi et al., 2016; Rolfo et al., 2015). In the northern slope of the Germanasca Valley, marble occurrences in the polycyclic basement are few and small in size (up to a few metres thick). Although several layers of marbles have been observed along the southeastern ridge of the Punta Muret, outcrop conditions did not allow to assess if these were due to several limestone layers interbedded in the pelitic protoliths, or if the repetitions are due to isoclinal folding.

### Serre Unit

The Serre Unit crops out discontinuously at the top of the tectonic stack of the northern Dora-Maira Massif, along its boundary with the overlying eclogite-facies ophiolites of the Orsiera-Rocciavrè Unit (Fig. 2). The Serre Unit reaches ~ 350 m in the Bourcet Valley and at the Col Clapier and thins out (only a few tens of metres thick) at Serrevecchio and at the Rocca Galmount, where it is directly in contact with the blueschist-facies meta-sediments of the Queyras Unit. The Serre Unit comprises several lithologies forming lenses and discontinuous layers of variable size that were tectonically juxtaposed.

*The Clapier Orthogneiss*

**Fig. 9:**
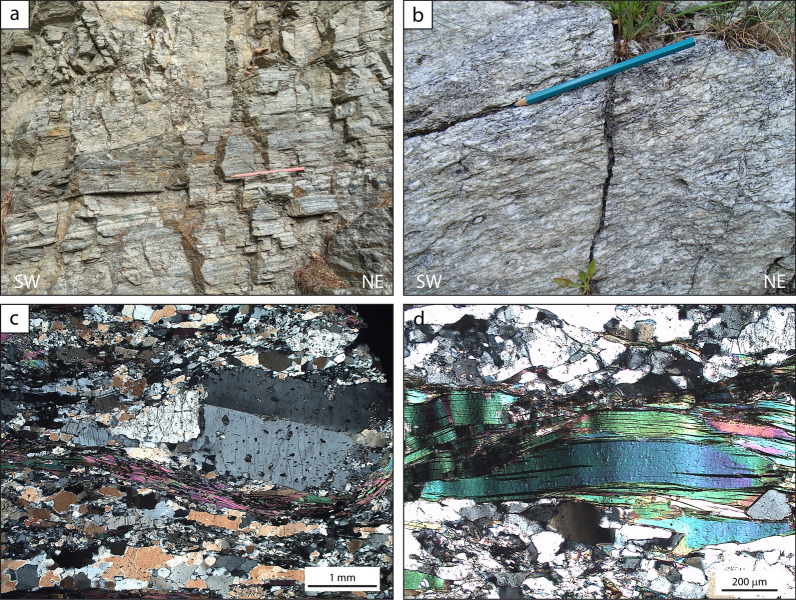
Field and microscopic aspect of the Clapier Orthogneiss. **a** A layered facies of the Clapier orthogneiss, with centimetre to decimetre scale layering related to differences in modal proportions of quartz, alkali feldspar and mica (44° 59′ 25.79′′ N–7° 06′ 20.23′′ E). **b** A homogenous facies with K-feldspar clasts and a well-developed foliation marked by muscovite (44° 59′ 2151′′ N–7° 06′ 15.38′′ E). Microphotographs show a twinned alkali feldspar (**c**), interpreted as a relict magmatic mineral, and deformed flakes of white mica (**d**), in a matrix of recrystallized quartz and albite

An orthogneiss, hereafter named Clapier Orthogneiss, generally occurs at the base of the Serre Unit and displays a thickness variable from a few metres to ~ 200 m (Fig. 2). The field aspect of the Clapier Orthogneiss is very heterogeneous (Fig. 9) and it mainly comprises augen gneisses (with alkali feldspar porphyroclasts up to 1.5 cm large), micro-augen gneisses (with porphyroclasts up to 6 mm in size) and fine-grained felsic leucocratic gneisses (lacking porphyroclasts). As a whole the alkali feldspar porphyroclasts are generally smaller and less abundant than in the orthogneisses of the Pinerolo Unit (Freidour Orthogneiss) and the Muret Unit (Granero and Muret Orthogneisses). The proportion of quartz, feldspars, white mica, titanite and tourmaline varies in different centimetre- to metre-thick layers.

*Micaschists*

Different types of micaschists occur in the Serre Unit, including silvery micaschists, ankerite-bearing micaschists, quartz-rich micaschists with large pseudomorphed sodic amphibole (up to 8 mm long) and minor garnet-chloritoid micaschists. Silvery micaschists are whitish in colour and consist solely of quartz and white mica. Ankerite forms millimetre-sized aggregates homogeneously dispersed within the micaschists or it concentrates in centimetre- to decimetre-thick layers and nodules. Layers of silvery micaschists and ankerite-bearing micaschists, from 5 cm to 2 m thick, are locally interlayered within the Clapier orthogneiss.

*Marbles and calcschists*

**Fig. 10:**
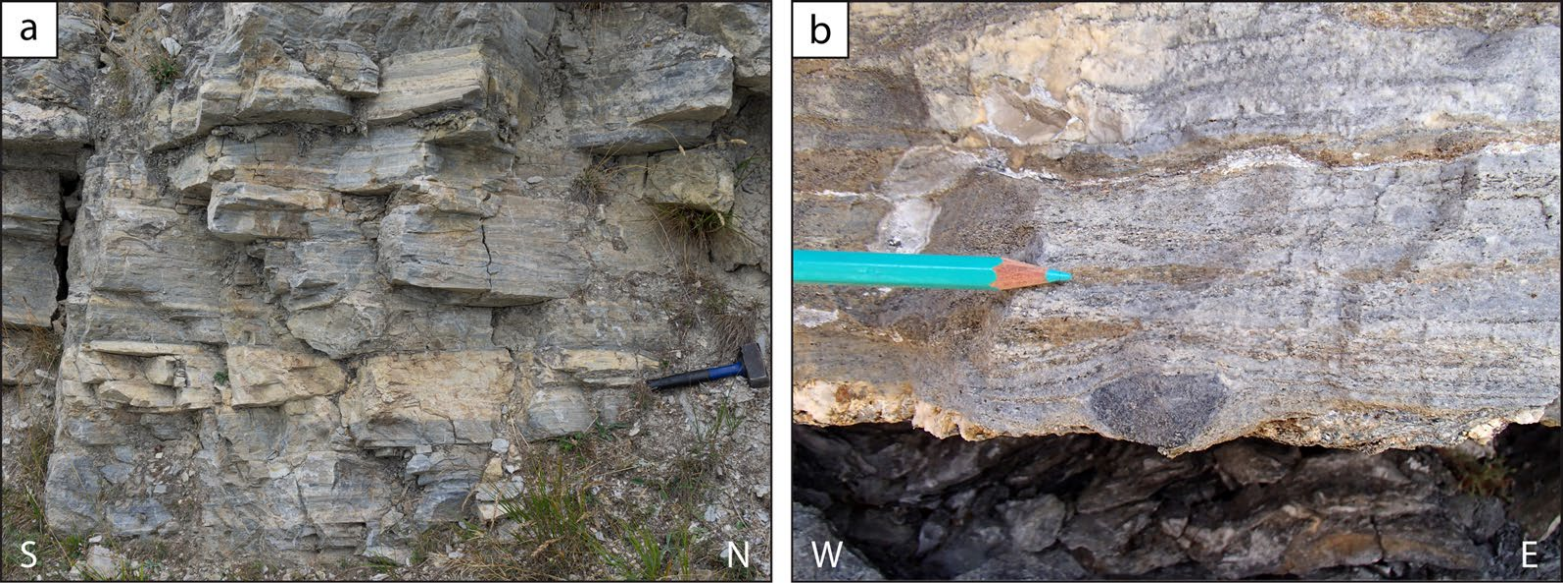
Field aspect of the layered dolomitic marbles (**a**, 44° 59′ 24.6′′ N–7° 06′ 08′′ E) and calcschist (**b**, 44° 59′ 20.96′′ N–7° 06′ 07.05′′ E) within the Serre Unit. The decimetre-thick layering in the dolomitic marbles (**a**) is a characteristic feature of most platform carbonates of Middle and Upper Triassic age in the Alpine belt. In the calcschist (**b**), a dolomitic clast, more resistant to the deformation than the calcite-rich matrix, suggests derivation from a carbonate breccia, potentially of Jurassic age (e.g. Pantet et al., 2020)

Dolostones, dolomitic and calcitic marbles and calcschists are a major and characteristic component of the Serre Unit (Fig. 10). Lenses of dolostones, ranging in size from 1 to 15 m, can be found in contact with micaschists, marbles and calcschists. Dolostones display millimetre- to decimetre-thick layers with alternated whitish and bluish colour and different proportion of dolomite and calcite (Fig. 10a). Marbles are found in moderate volumes (layers up to a few metres thick) and can be whitish, bluish or greyish in colour. Locally impure marbles are interlayered with minor carbonate-poor calcschist grey in colour (decimetre-thick). Rare centimetre-sized bluish lenses of dolomite, wrapped by the main foliation, are locally found within marbles. It is unclear if they derive from boudinaged dolomite layers or original dolomite clasts. Calcschists are commonly found in the Serre Unit and are generally rich in carbonate, sometimes displaying dolomite clasts (Fig. 10b). In carbonate-bearing rocks the foliation is defined by white mica in different modal amounts.

### Oceanic units

**Fig. 11:**
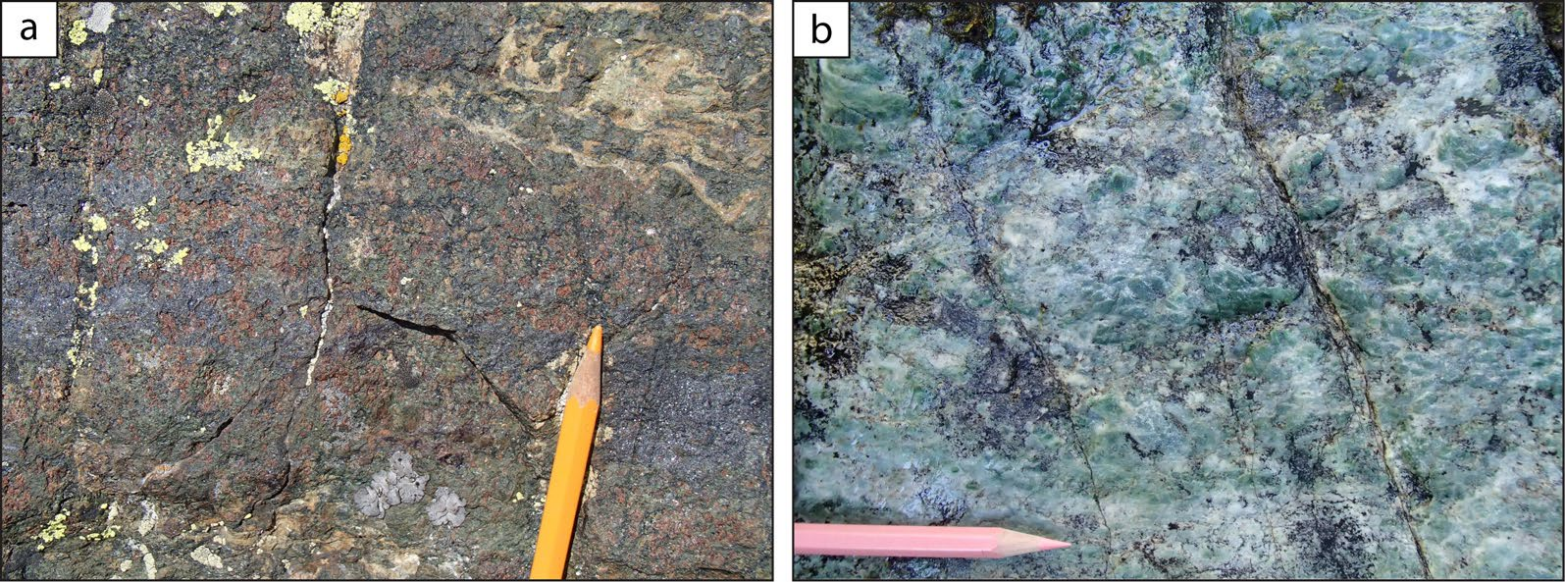
Lithologies of the Raccias Unit. **a** Characteristic feature of eclogite-facies Fe-Ti gabbro cropping out in the Punta Raccias area (44° 58′ 27.13′′ N, 7° 4′ 55.38′′ E) and of Mg–Al smaragdite gabbro (**b**) exposed close to Massello (44° 57′ 10.56′′ N, 7° 3′ 34.37′′ E). The eclogite (**a**) is essentially made of garnet, deep green omphacite, dark blue glaucophane and epidote. A large amount of rutile is seen at the microscope. The smaragdite metagabbro (**b**) displays bright green Cr-rich omphacite, topotactically replacing the magmatic clinopyroxene, in a foliated matrix consisting of clinozoisite, Mg-rich chlorite and tremolite/actinolite, with scarce rutile rimmed by titanite

Oceanic units are located on top of the nappe stack. Two main units are distinguishable. Firstly, serpentinites and eclogites, the latter deriving from FeTi-rich gabbros, crop out in the Punta Raccias area (Figs. 2, 11a). Another, very small, outcrop of eclogite-facies lithologies, namely serpentinites and smaragdite metagabbros, the latter deriving from Mg-rich gabbros, has been found close to Massello, along the bank of the Germanasca River (Fig. 11b). These lithologies characterize the internal, ophiolite-dominated, eclogite facies units in the Western Alps, better exposed in the Viso (Lombardo et al., 1978; Balestro et al., 2011; Locatelli et al., 2019), Rocciavrè (Pognante, 1985), and Susa areas (Ghignone et al., 2021). Secondly, in a large sector located west of the Dora-Maira Massif, the oceanic units are dominated by metasediments (calcschists), and contain only a few lenses of ophiolitic material (e.g. Monte Albergian, Corno et al., 2022). In addition, this sector displays a blueschist-facies Alpine overprint. It clearly represents the northern extension of the Queyras Unit, and is labelled accordingly. This twofold subdivision of the oceanic units is in line with previous work (Ballèvre et al., 1990; Agard, 2023), and does not need to be detailed here.

## Geochemistry and geochronology

A detailed geochronological study has been performed in three lithologies. Firstly, two samples of paragneisses along the Germanasca valley have been collected to the west of (i.e. structurally above) the Granero Orthogneiss. This area was previously attributed to the Faetto Complex, considered as the upward stratigraphic continuation of the Pinerolo Unit, and therefore of presumed Permian age (Borghi et al., 1985). The aim of our work, based on detrital zircon geochronology, is to assess the maximum age of deposition of the sedimentary protoliths of the paragneisses. Secondly, according to previous studies (Vialon, 1966; Borghi et al., 1984), the Granero Orthogneiss is intruding both the Pinerolo and the Faetto Complex. In our interpretation, the Granero Orthogneiss is the mylonitic sole of the Muret Unit, located immediately above the Chasteiran *UHP* Unit (Manzotti et al., 2022). The age of the magmatic protolith of the Granero Orthogneiss is therefore a key element for constraining the geometry of the nappe stack. Thirdly, the Clapier Orthogneiss, at the base of the Serre Unit, is also a prime lithological marker, considered by all authors as a potential Permian intrusion.

### Paragneisses in the “Faetto Complex”

**Fig. 12:**
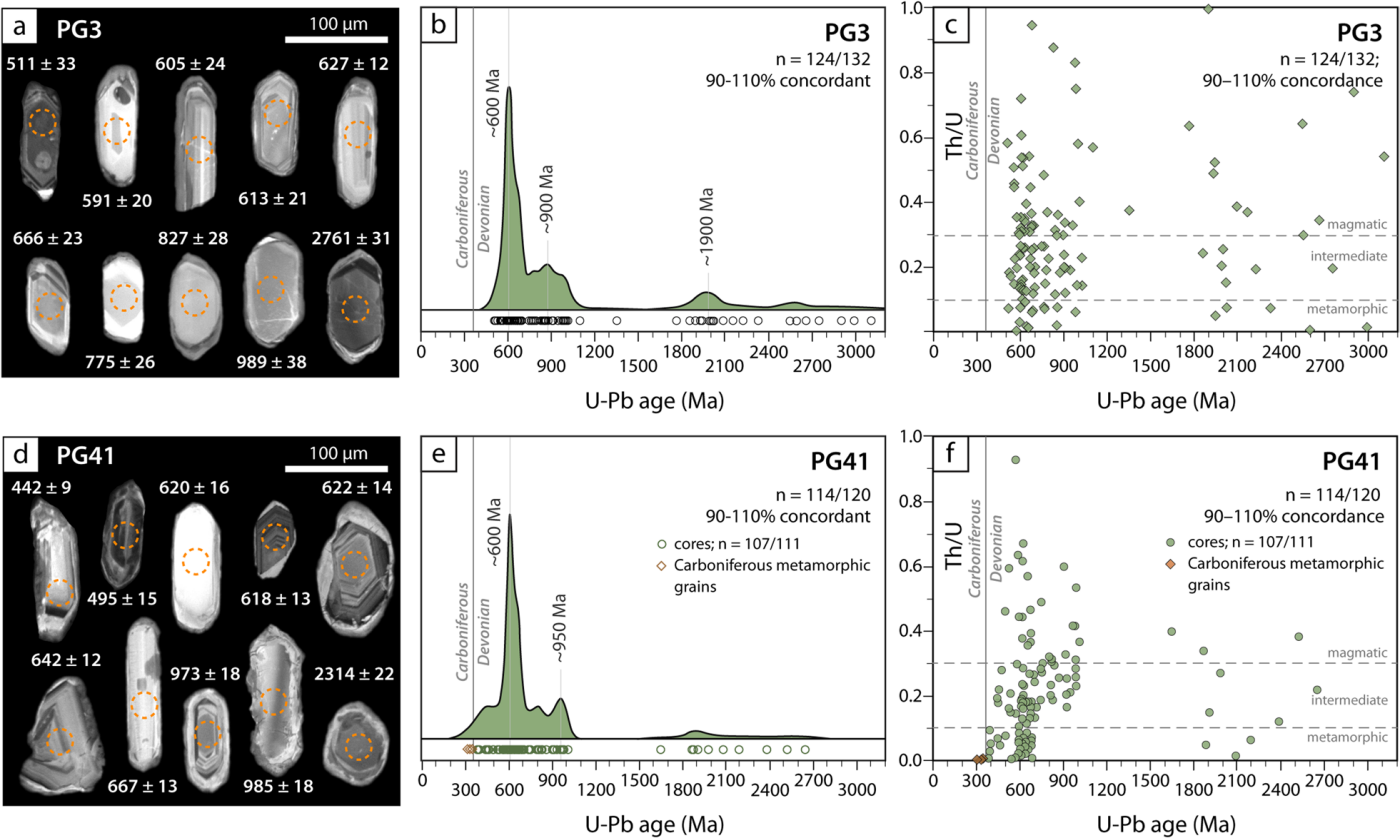
U–Pb zircon geochronology of paragneiss samples PG3 (**a**–**c**) and PG41 (**d**–**f**). **a, d** CL images of zircon. Dashed orange circles indicate the position of the LA-ICP-MS spots analysed for U–Pb ages. **b, e** Detrital zircon age distribution represented with Kernel density estimate (KDE). Individual dates are represented with empty cycles at the base of the diagram. **c, f** Th/U ratio versus age plot. Distinction between magmatic versus metamorphic zircon based on Th/U ratio is according to Teipel et al. (2004)

Samples PG3 and PG41 (Table 1; Fig. 2) are paragneiss collected in the Germanasca valley, close to the Rabbioso bridge (sample PG3, Fig. 8 and Table 1) and close to the contact with the underlying Granero Orthogneiss (sample PG41, Fig. 8 and Table 1). Sample PG3 (~ 35% white mica, ~ 35% quartz, ~ 10% chlorite, ~ 10% garnet, ~ 5% albite, and minor epidote, rutile, ilmenite, titanite, chloritoid and tourmaline) is poorer in albite compared to sample PG41 (~ 30% white mica, ~ 20% albite, ~ 20% quartz, ~ 10% chlorite, ~ 10% garnet, ~ 5% titanite, and minor epidote, rutile and tourmaline) and contains smaller garnet (PG3: ≤ 2 mm in size PG3; PG41: ≤ 6 mm in size). Zircon grains from the two samples display similar morphological features and thus are described together. Zircon crystals vary considerably in size and shape, ranging from 40 μm to 150 μm in length and from poorly elongated and rounded to highly elongated and subhedral (Fig. 12a, d). Zircon grains are slightly larger on average in sample PG41 than in sample PG3. The internal zoning shown by CL imaging is also variable, with most crystals displaying either an oscillatory zoning or being internally homogeneous. Most crystals are surrounded by a thin rim (mostly < 5 μm, but locally up to 10 μm in sample PG41) with a patchy and grey CL emission. A few crystals display a more complex zoning comprising multiple resorbed shells with different CL emission and internal zoning. In both samples, no systematic correlation was observed between age distribution and size and shape, nor between age distribution and internal zoning. This and the absence of strongly discordant crystals ensures that the age distribution is not biased by hydraulic sorting effects (e.g. selective grain loss during sample processing) and by inappropriate cut-off level for discordant ages in a detrital population (Malusà et al., 2013). Most magmatic grains show magmatic oscillatory zoning or few younger magmatic overgrowths surrounding older partially dissolved cores. Overall, in both samples, zircon grains vary considerably in U contents (PG3: 59–3104 ppm; PG41: 30–1622 ppm) and in Th/U ratio (PG3: 0.001–0.947; PG41: 0.001–0.927 in sample PG41), suggesting derivation from both magmatic and metamorphic sources (Hoskin and Schaltegger, 2003; Teipel et al., 2004). Metamorphic grains (Th/U<0.1) are either homogeneous or show inherited cores surrounded by overgrowths. No systematic correlation between age distribution and Th/U ratio has been observed (Fig. 12c, f).

In sample PG3, 132 zircon grains were analysed out of which 124 are concordant (Fig. 12b). The youngest magmatic concordant date is Cambrian (511 ± 33 Ma; Th/U = 0.58), whereas 12 concordant dates are younger than ~ 590 Ma, but do not define any age cluster. Indeed, if we calculate a concordia age using the dates younger than ~ 590 Ma we obtain a date of 556.7 ± 4.0 Ma with a high MSWD value (MSWD = 5.5, n = 12). The youngest age cluster is also the most abundant and yields a concordia date of 605.7 ± 3.1 (MSWD = 0.77, n = 22). This age cluster is part of a larger age population comprised between ~ 590 Ma and ~ 700 Ma (54 dates; Fig. 12b).

In sample PG41, 120 zircon grains were analysed, 114 out of which are concordant (Fig. 12e). The youngest magmatic concordant date is close to the boundary between the Ordovician and the Silurian (442 ± 9 Ma; Th/U = 0.19). The youngest magmatic age clusters comprise only very few dates (446.3 ± 4.4 (MSWD = 0.56, n = 4); 532.7 ± 5.9 (MSWD = 1.4, n = 4); 597.9 ± 4.2 (MSWD = 2.2, n = 7)). As in sample PG3, the largest age cluster yields a concordia age of 617.1 ± 2.6 Ma (MSWD = 1.4, n = 20), which is part of a larger population (46 dates; Fig. 12e) ranging from ~ 700 Ma to ~ 600 Ma. In sample PG41, seven metamorphic grains are younger than 400 Ma: four of them define a cluster at ⁓394 ± 7 Ma and three of them are Carboniferous in age and yield concordant dates of 343 ± 12 Ma, 329 ± 8 Ma, and 311 ± 9 Ma.

In both samples, the second major age population is relatively broad ranging from ~ 1050 Ma to ~ 750 Ma, with a peak at 950–900 Ma (34 and 27 dates in sample PG3 and PG41, respectively). A minor but significant age group clusters at ~ 1900 Ma. A few grains are older than 2000 Ma.

### Granero Orthogneiss

Three samples (OG34, OG36, and OG49, Table 1) from the Granero Orthogneiss were collected at three different locations (Fig. 2). All samples contain quartz (~ 45%), feldspars (~ 40%), white mica (~ 10%), and minor chlorite, and opaque. Sample OG49 contains some epidote and rare garnet. The size of the alkali feldspar porphyroclasts is generally ≥ 1 cm in sample OG34 (Fig. 7b) and < 1 cm in samples OG36 and OG49. Plotting of the whole-rock major element composition of the three samples in the R1R2 diagram (De La Roche et al., 1980) and in the TAS diagram (Le Maitre et al., 2002) indicates that their protolith was a granite or a rhyolite (Additional file 1: Fig S1). The alkali content is 8.9, 7.4 and 7.9 wt.% for sample OG34, OG36 and OG49, respectively (Additional file 1: Table S1). Sample OG34 and OG49 are moderately peraluminous (A/CNK = 1.22, 1.19, respectively) whereas sample GM36 is strongly peraluminous (A/CNK = 1.32; Additional file 1: Table S1).

**Fig. 13:**
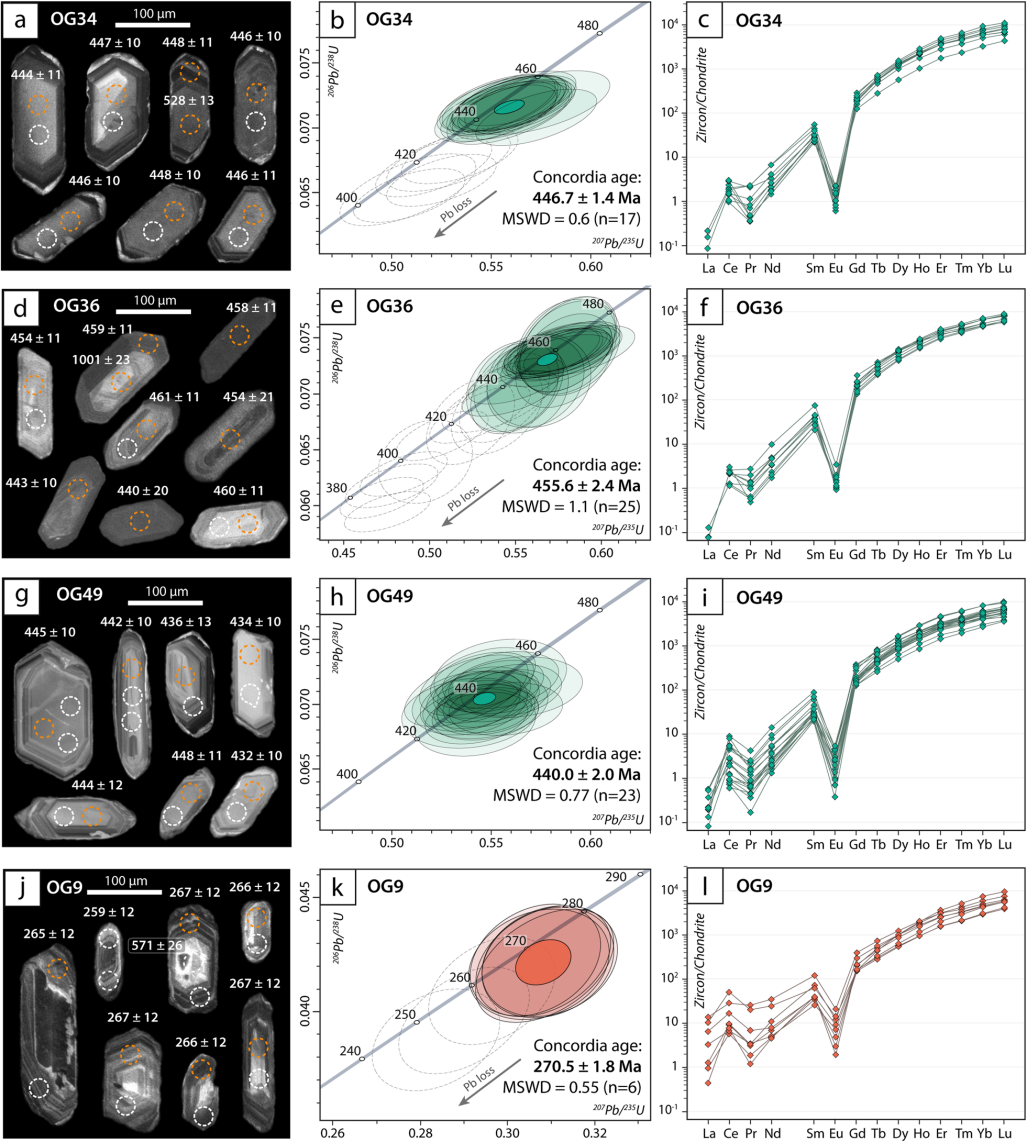
U–Pb zircon geochronology of orthogneiss samples OG34 (**a**–**c**), OG36 (**d**–**f**), OG49 (**g**–**i**) and OG9 (**j**–**l**). **a, d, g, j** Cathodoluminescence (CL) images of zircon. Dashed orange and white circles indicate the position of the LA-ICP-MS spots analysed for U–Pb ages and trace elements, respectively. **b, e, h, k** Concordia diagrams. Empty dashed ellipses represent dates excluded from the Concordia age calculation, as they likely suffered from Pb loss or mixing with metamorphic rims. **c, f, i, l** Trace element content of zircon. REE patterns are normalized to chondrite values (McDonough & Sun, 1995)

Zircon grains extracted from the three orthogneiss samples exhibit common characters and are hereafter described together. They are prismatic in shape and range from 80 to 150 μm in length. They are internally homogeneous or display a weak oscillatory zoning (Fig. 13a, d, g). A few crystals display inherited cores with prismatic shape. Very thin zircon overgrowths (< 5 μm) with bright CL emission are locally observed in sample OG34 and OG49 and, to a lesser extent, in sample OG36. Overgrowths display an embayed interface (at a scale < 5 μm) with the inner part of the zircon crystal. Zircon overgrowths have not been analysed due to their very small size. No difference in chemistry and dates has been observed between zircon crystals with homogeneous or oscillatory zoning.

Twenty-five analyses out of twenty-four different zircon crystals were performed for sample OG34. Th and U concentrations are in the range 16–172 ppm and 161–1949 ppm, respectively, with Th/U ratios mostly comprised between 0.02 and 0.10 with only a few analyses up to 0.37. Two concordant analyses obtained on inherited cores yield an apparent age around 520 Ma while the rest of the analyses provide concordant dates in the range 450–405 Ma. The main cluster (17 out of 23) defines a concordia date at 446.7 ± 2.6 Ma (MSWD (concordance + equivalence) = 0.6; n = 17; Fig. 13b). The position of the remaining 6 data is attributed to a slight Pb loss.

Forty-three analyses were performed on forty different zircon crystals in sample OG36 (Additional file 2: Table S2). Th and U concentrations are in the range 22–153 ppm and 203–4651 ppm, respectively, with Th/U ratios comprised between 0.01 and 0.10 and a few analyses up to 0.34. One grain yields an apparent age around 1 Ga. The remaining data plot in a concordant to discordant position (Fig. 13e). The older concordant cluster defines a concordia date at 455.6 ± 2.4 Ma (MSWD = 1.1; n = 25; Fig. 13e). The position of the remaining analyses can be attributed to slight to strong Pb loss during one (or more) event(s).

For sample OG49, twenty-three analyses out of 23 different zircon crystals were acquitted. Th and U concentrations are in the range 34–369 ppm and 215–1200 ppm, respectively, with Th/U ratios comprised between 0.02 and 0.20 and one analysis up to 0.40. They define a concordia date at 440.0 ± 2.0 Ma (MSWD = 0.77; n = 23; Fig. 13h).

Chondrite-normalized REE patterns in zircon for the three samples are enriched in HREE, with steep HREE pattern (Dy_N_/Yb_N_ = 0.17–0.22, 0.16–0.26 and 0.15–0.38 for sample OG34, OG36 and OG49, respectively), a strong negative Eu anomaly (Eu/Eu^*^ = 0.005–0.030, 0.006–0.016 and 0.005–0.039 for sample OG34, OG36 and OG49, respectively) and a slightly positive Ce anomaly (Fig. 13c, f, i; Additional file 2: Table S2).

### Clapier Orthogneiss in the Serre Unit

Sample OG7 and OG9 were collected from two separate outcrops belonging to the Clapier Orthogneiss in the Serre Unit (Table 1; Fig. 2). They are similar in mineral modal abundance (~ 50% quartz, ~ 35% feldspars, ~ 10% white mica, and minor chlorite, epidote and opaque) and display alkali feldspar porphyroclasts up to 1 cm in size (Fig. 9c). Plotting of the whole-rock major element composition in the R1R2 diagram and TAS diagram indicates that their protolith was a granite or a rhyolite (Additional file 1: Fig. S1). The alkali content is 7.8–8.2 wt.% and the A/CNK index is 1.1–1.2 (moderately peraluminous) in the two samples.

Zircon crystals were extracted from sample OG9. They are slightly less abundant than in the samples from the Granero Orthogneiss. They range from 70 to 200 μm in length, are prismatic in shape and display oscillatory zoning in CL (Fig. 13j). A few crystals contain an inherited core either with a prismatic shape and bright oscillatory CL emission or resorbed shape with dark patchy CL emission. Th concentrations in zircon are 54–326 ppm with one analysis at 663 ppm and one at 1364 ppm. U concentrations range from 494 to 3479 ppm and Th/U ratios range from 0.04 to 0.38. Four inherited cores yield apparent ages at 348 ± 13 Ma, 378 ± 14 Ma, 679 ± 24 Ma and 1945 ± 34 Ma, respectively with Th and U concentrations in the range 66–294 ppm and 448–729 ppm, respectively, and Th/U ratios of 0.11–0.46. The remaining data yield mostly discordant dates in the range 210–274 Ma. The oldest analyses (6 out of 22) are concordant and define a concordia date at 270.5 ± 1.8 (MSWD (concordance + equivalence) = 0.55; n = 6; Fig. 8k). Chondrite-normalized REE patterns are enriched in HREE, with steep HREE pattern (Dy_N_/Yb_N_ = 0.14–0.25), a negative Eu anomaly (Eu/Eu^*^ = 0.015–0.125) and a positive Ce anomaly (Fig. 13l; Additional file 2: Table S2).

## The Alpine architecture of the northern Dora-Maira Massif

### Reassessing the definition of the main units

In the following, we use the new field and geochronological data to reconsider the geometry of the tectonic stack along the studied section. According to previous authors (Wheeler, 1991; Sandrone et al., 1993; Gasco et al., 2011; Cadoppi et al., 2016) the tectonic stack of the northern Dora-Maira Massif consists of a lower monocyclic unit (the Pinerolo Unit) and an upper polycyclic unit with minor remnants of a Mesozoic cover. Some authors have also identified the Faetto Complex, a possible upward stratigraphic continuation of the Pinerolo Unit, therefore of presumed Permian age. Following Manzotti et al. (2022), our field work along the Chisone and Germanasca Valleys allowed us to identify four main tectonic units on the basis of their characteristic lithologies (detailed above) and of the recognition of tectonic boundaries (discussed below) (Figs. 2, 3).

The Pinerolo Unit is well-defined thanks to its characteristic meta-sedimentary successions. In particular, the occurrence of meta-conglomerates is a diagnostic feature, although they are not always present. Detrital zircon ages (Manzotti et al., 2016) and the occurrence of Permian meta-intrusives (Bussy and Cadoppi, 1996) have confirmed the presumed Upper Carboniferous age of the sediments (Novarese, 1895a, 1895b). As such, the Pinerolo Unit is indeed monocyclic, a statement consistent with the lack of pre-Alpine metamorphic parageneses.

The Chasteiran Unit mainly consists of a thin sheet (a few tens of metres thick; Figs. 2, 3) of graphite-rich chloritoid micaschists, structurally located above the Pinerolo Unit and below the Granero orthogneiss. Mapping the upper boundary of the Chasteiran Unit is therefore rather straightforward, but its lower boundary needs a clear distinction with respect to the meta-sediments belonging to the Pinerolo Unit. Due to their high modal amount of graphite, the chloritoid micaschists may be considered as the topmost part of the Pinerolo Unit. However, there are several observations indicating that the micaschists belong to a different unit.

Firstly, their lithology is distinctive. Indeed, chloritoid micaschists (with mica- and chloritoid-rich layers alternating with quartz-rich layers) are never found in association (interbedded) with meta-conglomerates or meta-sandstones. The most mica-rich meta-sediments from the Pinerolo Unit contain minute garnet grains and rare chloritoid included in garnet (only recognizable with a microscope). This allows the chloritoid micaschists of the Chasteiran Unit to be quite easily distinguished from the Pinerolo Unit.

Secondly, we would like to stress that the occurrence of graphite is not exclusive of the Pinerolo Unit and should be used with caution when trying to identify Pinerolo Unit meta-sediments in the field. Many basinal Palaeozoic sediments were shales deposited in anoxic, marine, environments which, upon metamorphism, will develop graphite-bearing assemblages. Such basinal sediments are not associated with conglomerates, whereas the close link between conglomerates and carbon-rich sandstones and siltstones is characteristic of the Late Carboniferous deposits in the Variscan belt. Therefore, the Chasteiran micaschists may derive from marine black shales of Early Palaeozoic (Silurian?) age, as suggested by Manzotti et al. (2022).

Thirdly, their metamorphism is also distinctive. Coesite has been found in the micaschists from the Chasteiran Unit, where the peak Alpine *P–T* conditions have been constrained at 27–28 kbar and 520–530 °C (Manzotti et al., 2022). By contrast, the Pinerolo Unit records lower *P–T* conditions, generally considered to have reached garnet-blueschist-facies in the northern Dora-Maira Massif (Borghi et al., 1985; Bousquet et al., 2012) or estimated at 20–23 kbar and 500–515 °C in the southern Dora-Maira Massif (Groppo et al., 2019). The existence of a gap in peak Alpine conditions between the higher *P* hangingwall rocks (belonging to the Chasteiran Unit) and the lower *P* footwall rocks (belonging to the Pinerolo Unit) implies that the two units are separated by a major tectonic boundary, here interpreted as a thrust (Fig. 2).

The Muret Unit consists of a pre-Carboniferous basement, with preserved relicts of the Variscan metamorphism and deformation. Low-strain volumes within the Muret Unit allow to describe in detail their pre-Alpine history (see Nosenzo et al., 2022). In contrast with the underlying Chasteiran Unit, in the Muret Unit peak Alpine *P–T* conditions do not reach the coesite stability field. They have been estimated at 21–22 kbar 530–560 °C in the Punta Muret area (Nosenzo et al., 2023). Similar values (at 18–20 kbar 515–520 °C) have been reported for polycyclic rocks located north of the studied area in the Susa Valley (Gasco et al., 2011). According to our data, both the Granero Orthogneiss and the Faetto Complex belong to the Muret Unit, and this deserves a specific comment.

The Granero Orthogneiss forms a thin (tens to hundreds of metres thick) sheet structurally above the Chasteiran Unit. In previous maps, it was considered an equivalent of the Freidour Orthogneiss and thus Permian in age and part of the Pinerolo Unit (Vialon, 1966; Borghi et al., 1984; Borghi and Sandrone, 1990). Most geologists would consider, on the basis of its well-developed augen structure, that the Granero Orthogneiss may derive from a porphyritic plutonic body. However, acidic volcanic or subvolcanic rocks may also be porphyritic, with feldspar crystals achieving a few cm in size (e.g. the Upper Cambrian to Lower Ordovician Ollo de Sappo volcanics; García-Arias et al., 2018; von Raumer and Stampfli, 2018). On the basis of our field observations, it is difficult to conclude whether the Granero Orthogneiss derives from a plutonic or a volcanic protolith. The observed heterogeneities in the distribution of the feldspar porphyroclasts and the compositional layering may result from different types of volcanic flows or different types of magma pulses within the plutonic body. Geochemistry of the three dated samples from the Granero Orthogneiss differs very slightly, with OG36 being more peraluminous than OG34 and OG49 (A/CNK = 1.32, 1.22 and 1.19, respectively). The major element content of the two samples is consistent with the protolith being either a granite or a rhyolite.

Zircon crystals from the three samples display typical magmatic features (e.g. Hoskin and Schaltegger 2003), such as oscillatory zoning, REE pattern with an enrichment in heavy REE, a negative Eu anomaly and a Ce positive anomaly (Fig. 8). The three samples yield an Ordovician to Lower Silurian emplacement ages for the magmatic protolith (446.8 ± 1.4 Ma, 455.6 ± 2.4 Ma and 440 ± 2.0 Ma for sample OG34, OG36, and OG49, respectively). This age difference may be a statistical artifact. Indeed, if we plot all data from the three samples in a single concordia diagram, we obtain a date of 446.9 ± 1.4 Ma (MSWD = 1.6, n = 64; Additional file 1: Fig. S2). Alternatively, the Granero Orthogneiss may consist of several magmatic bodies (either intrusive or extrusive) emplaced during a time span of several million years.

The Faetto Complex, located west (i.e. structurally above) the Granero Orthogneiss is essentially made of paragneiss associated with minor micaschist. Because some of these rocks contain graphite, and because the age of the Granero orthogneiss was not known, these sediments were grouped into the Faetto Complex, of presumed Permian age (Borghi et al., 1985). Our data allows to test this hypothesis. Detrital zircon grains from two samples of paragneiss of the “Faetto Complex” were analysed. The two studied samples show a similar age distribution. In contrast with the Upper Carboniferous meta-sediments of the Pinerolo Unit (Manzotti et al., 2016), the two paragneisses lack Carboniferous detrital zircons. The youngest magmatic zircon grains yield Cambrian (511 ± 33 Ma, sample PG3) and Silurian dates (442 ± 9 Ma, sample PG41). Dates younger than 590–600 Ma do not define any age cluster in sample PG3, or define only minor clusters with a limited number of dates in sample PG41, the youngest magmatic cluster being at 446 ± 4 Ma (n = 4). The most abundant cluster in both samples is Neoproterozoic (590–700 Ma) and provides concordia ages at 605.7 ± 3.1, MSWD = 0.77, n = 22 and 617.1 ± 2.6, MSWD = 1.4, n = 20 in sample PG3 and PG41, respectively. It follows that the main source providing detrital zircons in the Faetto Complex has a Late Neoproterozoic age. Another potential source of magmatic detrital zircon, defined by the cluster at ~ 446 Ma in PG41, could be Late Ordovician orthogneisses, like the Granero Orthogneiss.

Dates younger than ~ 400 Ma may result from different processes, such as (i) mixing between detrital cores and metamorphic rim, (ii) some Pb loss affecting older grains, and (iii) a minor detrital component from a younger source. With respect to the first hypothesis, during our analytical work, we carefully selected spots in order to avoid potential mixing between core and rim. In the second hypothesis, Pb loss may affect the source lithologies of the zircon or the sediments after their deposition, especially during a high-temperature (granulite-facies) metamorphism (e.g. Ewing et al., 2023). No petrographic or field evidence for a high-temperature metamorphism has been observed in the investigated paragneisses. In the Muret Unit, field and petrographic observations on micaschists suggests that this Unit did not experience partial melting and thermodynamic modelling indicate *P‒T* conditions of 6‒7 kbar ~ 650 °C during the Variscan orogeny (Nosenzo et al., 2022).

It therefore remains the third possibility, i.e. the presence of younger detrital grains. The four metamorphic dates defining a cluster at ~ 395 Ma can represent a minor source of detrital zircon grains. The three Carboniferous metamorphic dates (younger than 350 Ma) are interpreted as resulting from one or several metamorphic episodes associated with the Variscan orogeny and therefore are not used for defining the maximum age of sedimentation.

Interestingly, the two micaschist samples investigated by Nosenzo et al. (2022) in the Muret Unit display detrital zircon population similar to the ones observed in the Faetto Complex. Although a small number of zircon grains have been analysed by Nosenzo et al. (2022), their youngest cluster is Ediacaran in age (weighted average date of 598 ± 9 Ma, MSWD = 0.12, sample GM1) and Upper Ordovician (weighted average date of 453 ± 4 Ma MSWD = 0.72, n = 4, sample GM13), respectively.

To sum up, a conservative Late Neoproterozoic (~ 550 Ma) maximum age of deposition may be considered for the paragneisses of the Faetto Complex. However, considering the minor clusters at ~ 445 Ma and ~ 395 Ma, the maximum age of deposition may be much younger, i.e. Devonian in age. Further studies may eventually provide more compelling evidence for the occurrence of younger detrital grains. The huge difference in zircon populations between the sediments from the Pinerolo Unit (Manzotti et al., 2016) and the Faetto Complex (this study) excludes a Late Carboniferous or, even younger, Permian age for the deposition of the latter. We therefore propose to consider the Faetto Complex as part of the polycyclic Muret Unit.

The Serre Unit is the uppermost unit in the tectonic stack of the northern Dora-Maira Massif and it is discontinuously exposed at the contact with the oceanic units (Figs. 2, 3). In the Serre Unit, the Clapier Orthogneiss and associated meta-sediments (e.g. ankerite-bearing micaschist) have long been interpreted as an acidic volcanic and volcaniclastic sequence and assumed to be Permian in age (“*porphyroide arkosique*”, Vialon, 1966; Michard, 1967). Our field observations, such as the interbedding of the Clapier Orthogneiss and the meta-sediments and the strongly heterogeneous aspect of the orthogneiss in terms of mineral modal proportions and abundance and size of alkali feldspar porphyroclasts (Fig. 9), support this genetic interpretation. It is not an easy task, sometimes, to ascertain whether a given orthogneiss layer derives from a volcanic flow or an arkosic sediment eroded from a magmatic rock and redeposited in proximity to the source. The major element composition of the Clapier Orthogneiss is consistent with the protolith being a rhyolite. Zircon crystals display typical magmatic features, such as oscillatory zoning, enrichment in HREE, negative Eu anomaly and positive Ce anomaly. Zircon dating allows to establish a Permian age (270.5 ± 1.8 Ma) for the emplacement of the magmatic protolith of the Clapier Orthogneiss or for the magmatic source-rock of the arkosic sediments.

The Serre Unit consists of tectonically juxtaposed slices of Permian volcanic and volcanoclastic products and of dismembered Mesozoic cover. Although Permian rocks are generally found at the base of the unit and Mesozoic rocks are mostly found in the upper part of the unit, the primary stratigraphic relations between the different lithologies have been largely lost due to the Alpine reworking.

### Reassessing the boundaries of the main units

According to our map, two main tectonic boundaries are found within the Dora-Maira Massif. The most important one separates the Pinerolo Unit in the footwall from the Muret Unit in the hangingwall, and is marked by the location of the Chasteiran micaschist and the Granero Orthogneiss, two thin sheets that can be followed along this boundary for about 15 km (Figs. 2, 3). Another major tectonic boundary separates the Muret Unit in the footwall from the Serre Unit in the hangingwall. In addition, some late, ductile to brittle, faults cut across the entire nappe stack. Although the displacement along these late faults is probably minor (a few hundred metres at most) compared to the early ductile shears (several tens of kilometres), they have a marked influence on the map pattern. Both types of boundaries are briefly described below.

#### The contact at the base of the Muret Unit

The two largest tectonic units in the northern Dora-Maira Massif are the Pinerolo Unit in the footwall and the Muret Unit in the hangingwall (Figs. 2, 3). These Units are separated by two thin sheets of distinctive lithologies, namely the chloritoid schists from the Chasteiran Unit and the Granero orthogneiss.

In the Chasteiran micaschist, the main, regional, foliation is parallel to boundary with the Pinerolo Unit below and the Granero Orthogneiss above. Detailed examination at outcrop- or thin section-scale reveals that this main regional foliation is a composite foliation S_1–2_, resulting from a pervasive, isoclinal, folding at mm to cm-scale of an earlier fabric S_1_ (Manzotti et al., 2022). The microfolding is contemporaneous with or slightly postdating the latest increment of garnet growth. This indicates that the main, regional, foliation developed during exhumation of the Chasteiran Unit, at *P* at about 10–15 kbar, i.e. in the albite stability field.

The Granero Orthogneiss displays a pervasive mylonitic foliation which is parallel to the boundaries of the orthogneiss body, generally gently- to moderately dipping to the West (Fig. 3). Evidence for folding of former aplitic dykes, and of a relict early foliation, is locally observed (Fig. 7b). The mylonitic foliation is associated with a very strong EW-trending stretching lineation defined by the elongation of quartz and feldspar ribbons and by the alignment of white mica (Fig. 7c). The main foliation is therefore contemporaneous with the late-stage folding in the Chasteiran micaschists. Large muscovite grains, possibly developed at peak *P*, display undulose extinction and are frequently partly recrystallized. *UHP* relicts are not observed in the Granero Orthogneiss (e.g. polygonal quartz aggregate and palisade quartz derived from the transformation of coesite; Compagnoni and Rolfo, 2003) and thus this lithology is considered as part of the Muret Unit and not of the Chasteiran Unit (Fig. 2). However, we cannot exclude that pervasive deformation may have obliterated the *UHP* relicts, and thus that the Granero Orthogneiss may be part of the Chasteiran Unit instead.

**Fig. 14:**
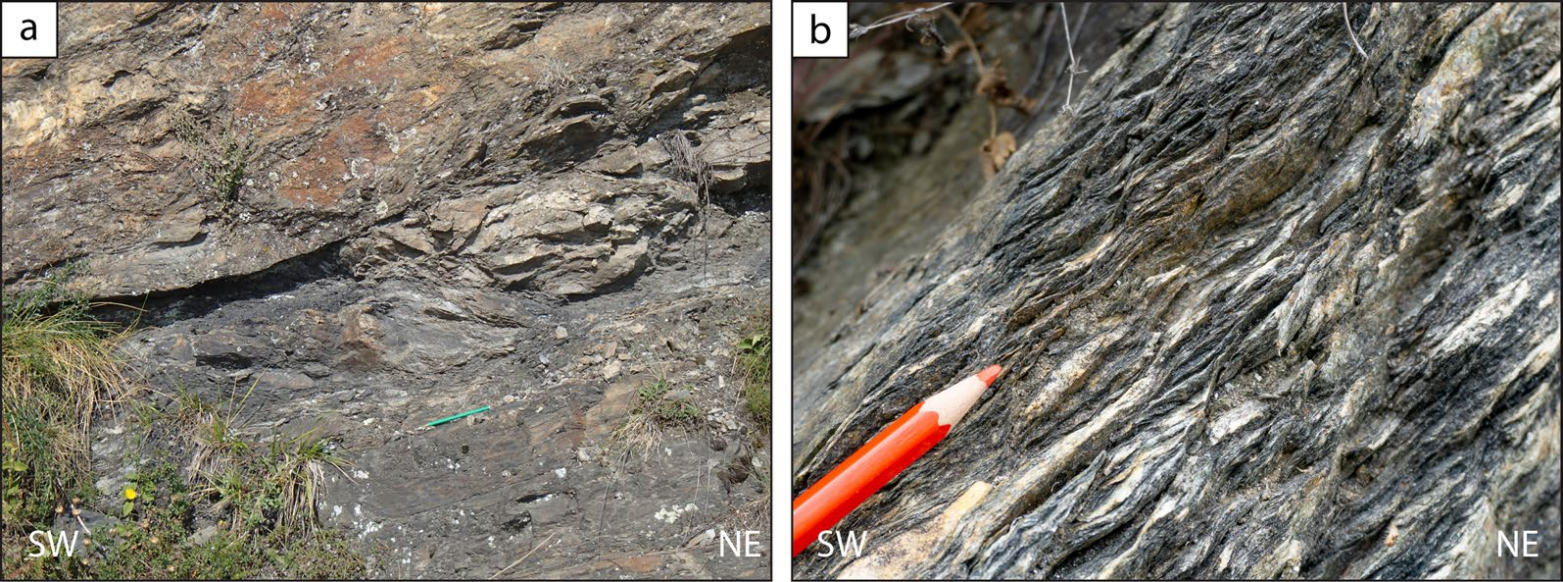
Shear criteria  in rocks in the northern Dora-Maira Massif. **a** Brittle to ductile deformation with top-to-the-W detachment layer in graphite-rich meta-siltstones from the Pinerolo Unit (44° 59′ 30.6′′ N–7° 06′ 59.06′′ E). **b** Top-to-the-W ductile shear bands in micaschists and calcschists close to the contact between the Muret Unit and the Serre Unit (44° 55′ 30.5′′ N–7° 03′ 34.2′′ E)

Shear criteria are not ubiquitous in the studied area and this is possibly due to the intense flattening. However, evidence of top-to-the-W or NW non-coaxial deformation has locally been observed in the Chasteiran Unit, such as shear bands, and in the structurally upper part of the Pinerolo Unit, such as detachment layers localized in graphite-rich meta-siltstones (Fig. 14a). Shear bands in the Chasteiran Unit are not observed when micaschists are crenulated. Given the very limited thickness of the Chasteiran Unit, the poor outcrop conditions and the locally intense crenulation, we cannot document, at this stage, if the strain increases in intensity towards the upper and lower boundaries of the unit. However, at the contact between the Chasteiran Unit and the underlaying Pinerolo Unit, we locally observed a graphite-rich layer, a few decimetres thick. Abundant graphite along this tectonic contact may have acted as a zone of weakness, accommodating displacement during thrusting.

#### The contact at the base of the Serre Unit

The Serre Unit as a whole is also characterized by a pervasive ductile deformation. With regards to its relations with the underlying units, two sectors need to be distinguished.

In the southern part of the studied area (from Prali to Massello), where the contact between the Dora-Maira Massif and the calcschists from the Piemonte-Liguria ocean is running almost North–South, the Serre Unit appears to be discontinuous at map scale (Fig. 2), and displays an increasing strain towards the base of the Unit. For example, in Serrevecchio, the boundary with the underlaying Muret Unit is marked by a ductile shear zone (a few metres thick) with slices of micaschists, calcschists, orthogneisses and serpentinite schists. These lithologies display a strong mylonitic foliation with abundant top-to-the-W shear bands (e.g. in Serrevecchio; Fig. 14b).

**Fig. 15:**
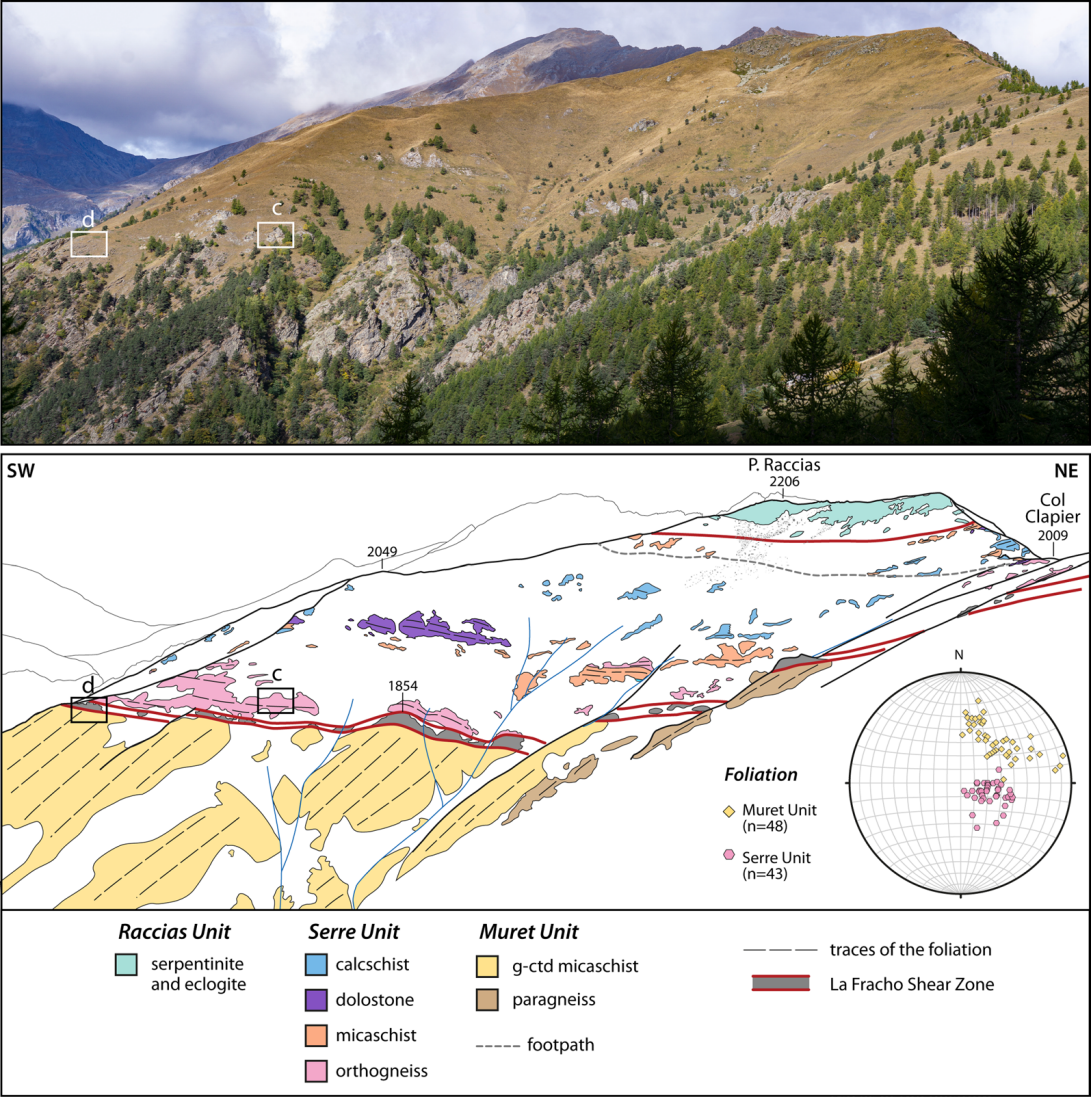
Panorama of the SE slope of the Col Clapier and Punta Raccias. The main foliation in the Muret and Serre Units displays a different average orientation (see the lower hemisphere Schmidt (equal-area) projection of the main foliation). The tectonic boundary between the Muret and Serre Units is marked by zone of intense deformation (i.e. La Fracho Shear Zone) characterised by CS structures. The white boxes labelled** c** and** d** refer to the photographs of Fig. 17

**Fig. 16:**
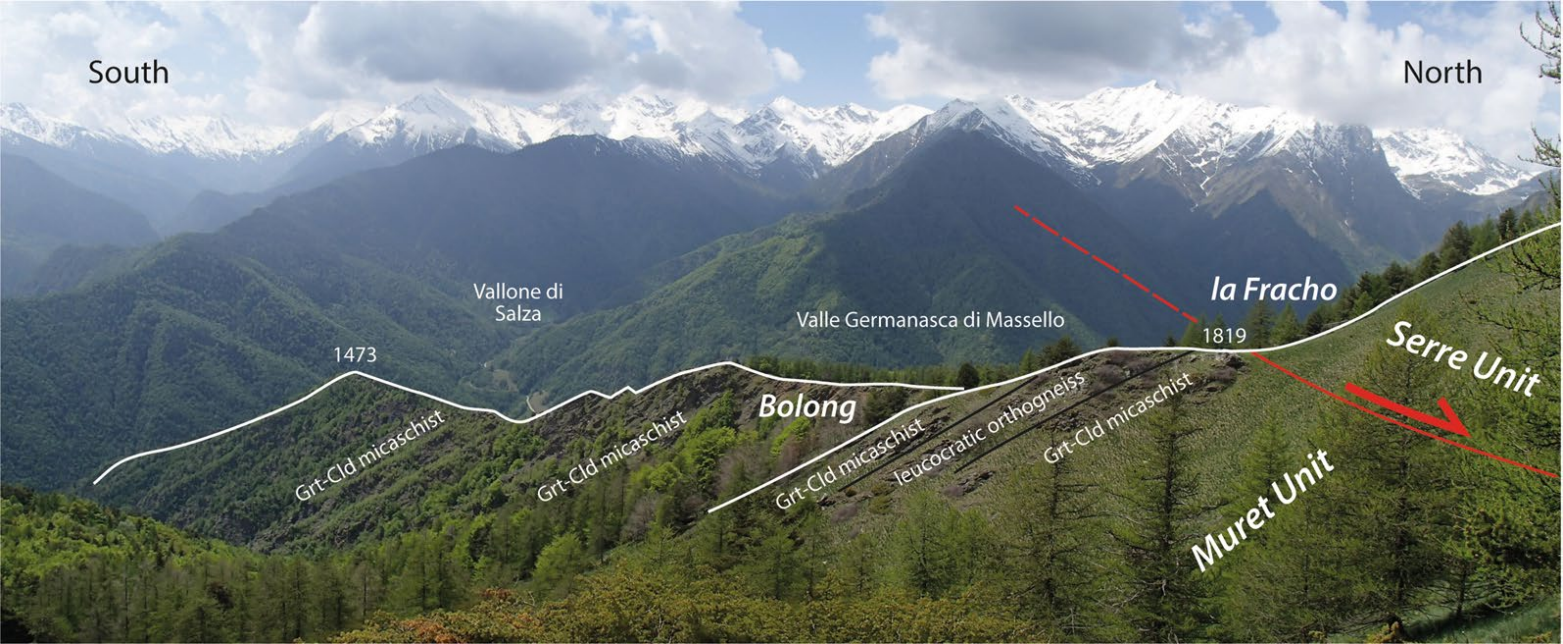
Panorama of the crest south of the Punta Raccias, displaying the south-west dipping lithologies in the Muret Unit abruptly cut by the tectonic contact at the base of the Serre Unit, (grassy slopes covering some orthogneisses and calcschists on the right). The location of panorama is displayed on Fig. 2

**Fig. 17:**
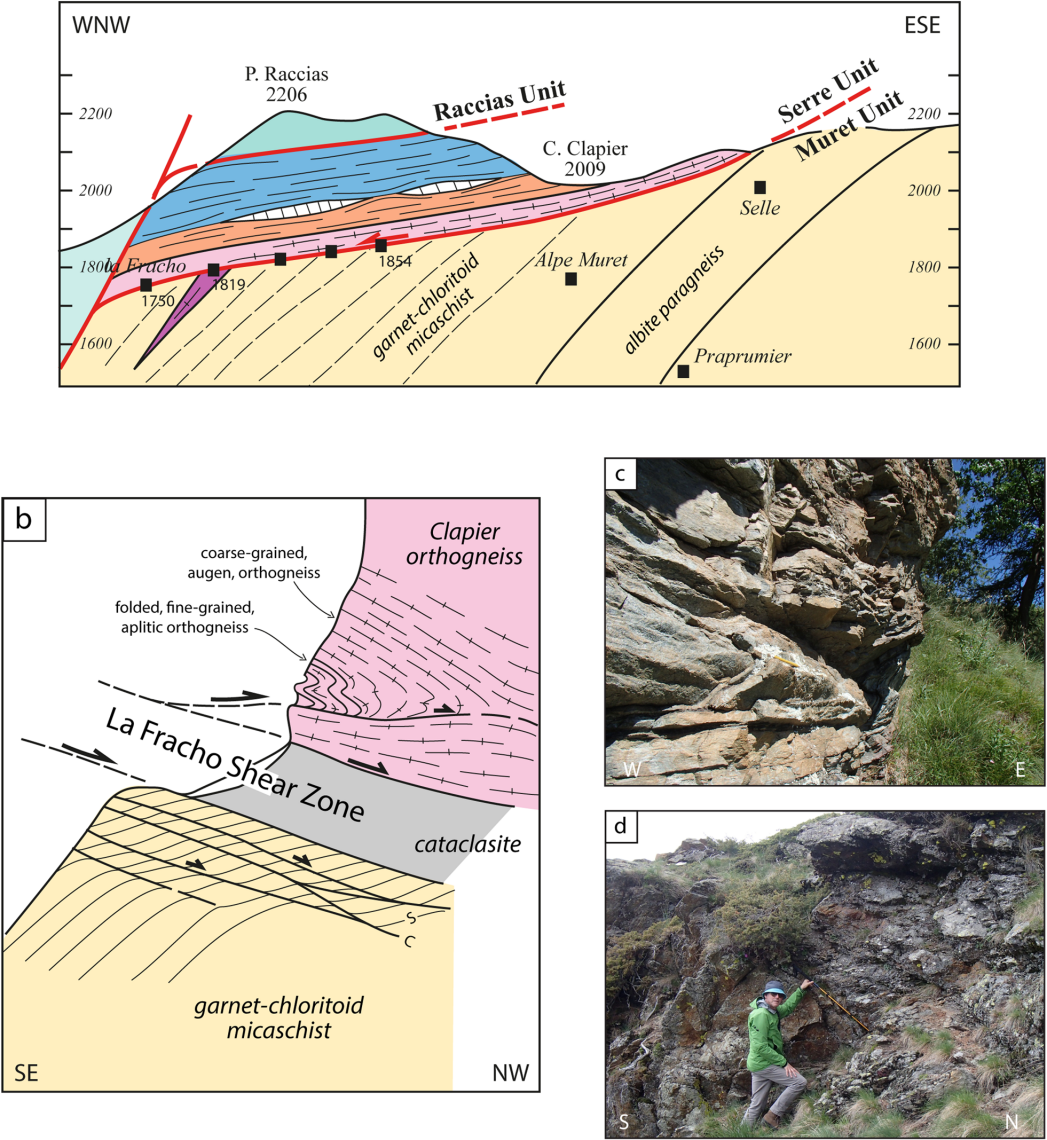
Simplified cross-section (**a**) and kinematic indicators along the La Fracho Shear Zone (**b**). The location of the outcrops best displaying asymmetric folds in the hangingwall (**c**) and CS strcutures in the footwall (**d**) is shown on Fig. 15

In a large northern sector, extending from the Germanasca Valley (Massello) to the Chisone Valley (north of Balma) (Fig. 2), the most important character of the contact between the Muret and Serre Units is the large difference in strike and dip between the structures in both Units (Figs. 3, 15, 16, 17). This has been recognized previously in the studied area, and interpreted as recording an unconformity possibly due to a late Variscan (Saalian) episode (Vialon, 1961 and 1966). According to this author, the base of Serre Unit is made of arkosic sandstones and conglomerates, deposited unconformably on top a Variscan basement. However, this interpretation is challenged by two important observations which suggest that the discordance is the result of Alpine tectonics rather than a post-Variscan stratigraphic unconformity. Firstly, the arkosic sandstones of Vialon (1961) are made in a large proportion of strongly deformed porphyritic volcanics, and their products reworked sub-contemporaneously, grouped together in this paper under the heading Clapier Orthogneiss. As anticipated correctly by Vialon (1961), this material is indeed of Permian age, now dated at 271 ± 2 Ma (Fig. 13k). We have not been able to identify meta-conglomerates that would mark the start of the Permian sedimentation on top of the Variscan basement. Secondly, and most importantly, the main foliation in the Muret Unit is an Alpine *HP* foliation (marked by phengite and chloritoid), and not a Variscan foliation (the low-strain domain of the Punta Muret is, in fact, located further east—see Fig. 2).

The best place, in terms of exposure, to study this contact is located south of the Punta Raccias, between La Fracho and the Col Clapier (Figs. 15, 16, 17), as recognized by Vialon (1961 and 1966). There, the boundary is marked by a few metres-thick ductile–brittle shear zone (hereafter referred to as La Fracho Shear Zone). The orientation of the main foliation (and the parallelized lithological boundaries) is discordant between the Muret Unit and the Serre Unit, being steeply dipping (45–50° on average) to the SW in the former and gently dipping (20–25°) to the west in the latter (Figs. 15 and 16). The shear zone is oriented parallel to the main foliation within the Serre Unit and crosscuts the main foliation within the Muret Unit (Figs. 15, 16, 17). The topmost part of the Muret Unit is characterized by the development of CS structures, with shear planes parallel to the main contact cutting across and deforming the regional foliation in garnet-chloritoid micaschists (Fig. 17). The shear sense displayed by the CS structures is unambiguously top to the NW. At the base of the Serre Unit, the main regional foliation displayed by the Clapier Orthogneiss is deformed by asymmetric, open, decimetre to metre folds (Fig. 17). The latter show axial plane slightly dipping to the NW with fold axes about N60°. The asymmetry of the folds and their association with foliation-parallel shear zones also indicate a top to the NW sense of shear. We conclude that the contact between the Muret and the Serre Units is a late (post-metamorphic) Alpine shear zone, developed at the ductile to brittle transition, displaying a top-to-the-NW displacement of the hangingwall with respect to the footwall.

#### Late, brittle, extensional faults

A set of steeply-dipping, brittle, faults with an WSW-ENE strike, offset the trace of the Granero Orthogneiss by ~ 1.5 km, and will be hereafter called the Trossieri Fault (Fig. 2). The dextral offset of the Trossieri Fault is only apparent, due to the westward dip of the reference marker bed (in this case the Granero Orthogneiss). The true displacement along the fault plane is normal, with the northern block being downfaulted with respect to the southern block. This fault explains why the Granero Orthogneiss is abruptly ending along the Germanasca valley close to Trossieri (Fig. 2), as reported in previous maps (Mattirolo et al., 1913 and 1951; Vialon, 1967; Borghi et al., 1984; Sandrone et al. 1993). The Trossieri Fault itself is very rarely seen, with one notable exception (44°55′42.16′′ N, 7°07′08.88′′ E). In the area close to the fault, the earlier ductile fabrics are overprinted by a set of conjugate microfaults and semi-brittle shear bands.

Normal brittle faults with a similar orientation and displacement, indicating an almost NS extensional episode, are described west of the study area, where they are quite common (e.g. Sue and Tricart, 2003). It is possible that this set of faults is much more easily detected in the oceanic units from the Viso and Queyras Units, with extensive outcrops above the tree line, than in the forested Dora-Maira area. Alternatively, it is also possible that the previous lack of recognition of these faults in the Dora-Maira Massif reflects that their density decreases from the core of the mountain belt to the Po plain.

## A brief summary of the geological history of the Dora-Maira Massif

The rocks from the Dora-Maira Massif preserve a wealth of information related to their geological history, since the Early Palaeozoic to the late Alpine evolution. Some of this information is only briefly repeated here, having been already detailed for the pre-Alpine history in a previous paper (Nosenzo et al., 2022).

### Pre-Carboniferous history

The pre-Carboniferous history, before the deformation and metamorphism associated with the Variscan orogeny, is recorded in the Muret Unit by the silico-clastic and carbonate deposition and by the Ordovician magmatism, i.e. the Muret orthogneiss at ~ 442 Ma (Nosenzo et al., 2022) and the Granero Orthogneiss in one or several pulses close to the Ordovician–Silurian boundary (this study). The Ordovician granitic and rhyolitic magmatism is well known all over the southern part of the Variscan belt from northern Spain (Talavera et al., 2013), Western France (Ballèvre et al., 2012), to the Pyrenees (Navidad et al., 2018), Sardinia (Cruciani et al., 2013) and the Alps (Bergomi et al., 2017; Chen et al., 2023; Gilotti et al., 2023). Although part of this magmatism is older than the ages we have obtained for the Granero Orthogneiss, and also for the Muret Orthogneiss, similar Upper Ordovician ages are recorded in the Pyrenees, the Montagne Noire and Sardinia (see Stephan et al., 2019, and Álvaro et al., 2020, for a review). The diversity of metasediments in the Dora-Maira polycyclic basement reflects a long history, partly deciphered thanks to the detrital zircon geochronology. The protoliths of the paragneisses from the Muret Unit (essentially consisting of wakes) indicate erosion of a Late Neoproterozoic source (~ 600 Ma old), with a potential minor component of slightly younger ages. The same source is involved during sedimentation of the pelites from the Muret Unit (Nosenzo et al., 2022). Similar ages for detrital material have been found in the external part of the Briançonnais Zone (Thiéblemont et al., 2023). The exact age of the carbonate sedimentation is still uncertain (for a discussion, see Nosenzo et al., 2022).

### Carboniferous history

The Variscan metamorphism, and associated ductile deformation, is well recorded in the Muret Unit and indicates involvement into the Variscan continental collision and associated crustal thickening (Nosenzo et al., 2022). More details have been emphasized in this study concerning the post-Variscan sedimentation in the Pinerolo Unit. Because of their characteristic lithology and their detrital zircon record, the Carboniferous age of the Pinerolo Unit meta-sediments is well established (Manzotti et al., 2016), and they have been correctly compared to the fossiliferous sediments of the Zone Houillère, in the most external part of the Briançonnais domain. However, there is a major difference between the two areas in terms of Alpine deformation, which is much more intense in our study area. Certainly, the thickness of the layers described in these sequences and reported in Figs. 4 and 5 does not correspond to the original thickness of the sedimentary beds, due to subsequent Alpine reworking. In particular coal layers may have lost a considerable volume during the transformation of coal (density of 1.1–1.5 g/cm^3^) into graphite (density of 2.2 g/cm^3^). In addition, graphite-rich layers were often strongly sheared during the Alpine deformation due to their weaker rheology, displaying disharmonic folding. However, likewise in other strongly deformed and metamorphosed meta-sediments (e.g. Banks, 2007), some inferences about their depositional environment can be made.

The Ponte Raut successions, characterized by the abundance of graded bedding and cross bedding and the presence of laterally discontinuous conglomerate layers, are best interpreted as fluvial successions. The source area was probably close to the depositional area, given the relatively low textural and compositional maturity (Nichols, 2023). Although quartz veins provided the dominant material for the conglomerates, some magmatic and metamorphic rocks were also eroded, as nicely displayed by the pebbles of felsic gneiss and rare pebbles of garnet-bearing micaschists. Additionally, we interpret the very flattened pebbles of black schist as reworked clay chips, once again pointing toward an environment with local mud deposits, possibly in temporarily desiccated river flood-plains.

The Bourcet successions can be considered as resulting from a cyclic sedimentation, with fluvial coarse-grained deposits invading ponds or marshes into which fine-grained mud and organic matter was slowly accumulating. This type of cyclicity has been described in many late Carboniferous basins and is due to the complex interplay between auto- and allo-cyclic processes (see discussion in Fielding, 2021).

The Pons successions need a rather low-energy environment of deposition, such as a temporary lake into which rivers were episodically discharging sandy material. The measured section should have been located in a rather distal part of the river delta into the lake, because only material no coarser than sand was interrupting the clay, silt and organic matter deposition.

We have not been able to map the three types of successions described above, because of discontinuity of outcrops and because some transitional types exist. In addition, we suspect the existence of east–west trending kilometre-scale folds within the Pinerolo Unit. It is therefore not possible, at this stage, to document if the occurrence of the different types of successions indicate a transition in time or in space (or both) within the late Carboniferous basin.

To conclude, the Pinerolo sediments have been deposited in a late Carboniferous basin surrounded by reliefs made of rocks strongly deformed and metamorphosed during the Variscan orogeny that provided abundant detrital material to the basin. This material was transported and deposited by fast-flowing rivers (Ponte Raut succession), that episodically flooded their alluvial plains (Bourcet succession) or invaded ephemeral lakes (Pons succession). This is quite typical of all “intramontane” basins developed on top of the collapsing Variscan belt during the Late Carboniferous (e.g. Ballèvre et al., 2018).

### Permian history

Accepting the Carboniferous age of the Pinerolo meta-sediments and considering the Permian age of the orthogneisses found in the Pinerolo Unit (Bussy and Cadoppi, 1996), we should expect to find evidence of primary intrusive relationships of the granitic and dioritic magmatic bodies into the Carboniferous sediments. From sample to outcrop scale and map scale, the contact between the orthoderivates and the meta-sediments is parallelized to the main Alpine foliation. Decisive field evidence that the magmatic bodies were intruded into the sediments of the Pinerolo Unit has not been found yet. However, one can note that Novarese (1895b) reported the occurrence of cm-sized aggregates consisting of white mica ± chloritoid ± chlorite in graphite-rich schists close to the Freidour Orthogneiss (lower Sangone Valley) and dioritic bodies (lower and middle Chisone Valley). Novarese (1895b) interpreted them as pseudomorphs after andalusite derived from the contact metamorphism associated with emplacement of the magmatic bodies.

Permian acidic and intermediate magmatism (both intrusive and extrusive) is widespread in the Dora-Maira Massif and, in general, in the Briançonnais Domain, whereas it is essentially absent in the External Massifs, where a Carboniferous magmatism is recorded instead (Ballèvre et al., 2018, 2020, and references therein). During the Permian, high temperature metamorphism and partial melting was occurring in the lower crust, whereas melt was emplaced in the upper crust, with volcanism and opening of fault-bounded basins at the surface. The Permian volcanic and volcaniclastic rocks in the Serre Unit represents such upper crustal products. This geodynamic event records the collapse of the Variscan belt, possibly concurrent with the activation of transcurrent faults such as the East Variscan Shear Zone (Ballèvre et al., 2018; Elter et al., 2020; Simonetti et al., 2020; Fréville et al., 2022, Vanardois et al., 2022; Bühler et al., 2023). The new data provided in this study are consistent with the palaeogeography as depicted in Ballèvre et al. (2018).

### Mesozoic history

Given the intensity of the Alpine deformation, the Mesozoic history can only be rather poorly documented. In the Serre Unit, dolostones, marbles and calcschists, are generally found on top of the Permian magmatic and sedimentary rocks. This carbonate-bearing succession is characteristic of the Mesozoic cover (Fig. 10). Although we have not been able to find fossils in the studied area, some have been found in similar lithologies further south, in the Maira Valley (Franchi, 1911; Michard, 1967), and north, in the Susa Valley (Franchi 1911; Marthaler et al., 1986). By comparison with the “classical” Mesozoic successions in the External Briançonnais (e.g. Ellenberger, 1958) or with the “Pre-Piemonte-type” successions in the Internal Briançonnais (including the Val Maira-Sampeyre Unit and Val Grana Unit along the south-eastern margin of the Dora-Maira Massif; Balestro et al., 2022; Michard et al., 2022; Pantet et al., 2022; Dana et al., 2023), dolostones are typically considered Middle to Upper Triassic in age and limestones and calcschists can be considered Jurassic and Cretaceous in age, respectively (Fig. 10).

This sedimentation is the result of Triassic crustal extension, subsequent Jurassic rifting of Pangea with development of the Briançonnais passive margin, and final oceanic spreading starting from the Mid- to Late Jurassic (e.g. Lemoine and Trümpy, 1987; Cordey and Bailly, 2007). It has been proposed that the units of the Dora-Maira Massif constituted different crustal fragments already during the Mesozoic as part of the extended Briançonnais palaeomargin (Ballèvre et al., 2020; Bonnet et al., 2022). In such a hypothesis, the different units were separated by normal faults or, in case they represented extensional allochthons, by exhumed mantle or oceanic crust. Tectonic slices of serpentinites and meta-basites are, in fact, found along the boundary between two units (the Rocca-Solei Dronero Units) in the southern part of the massif, where they constitute the Valmala-Piasco Shear Zone. This hypothesis may also be valid in the northern Dora-Maira Massif, being supported by the occurrence of serpentinites at the contact between the Muret and the Serre Units.

### Alpine history

**Fig. 18:**
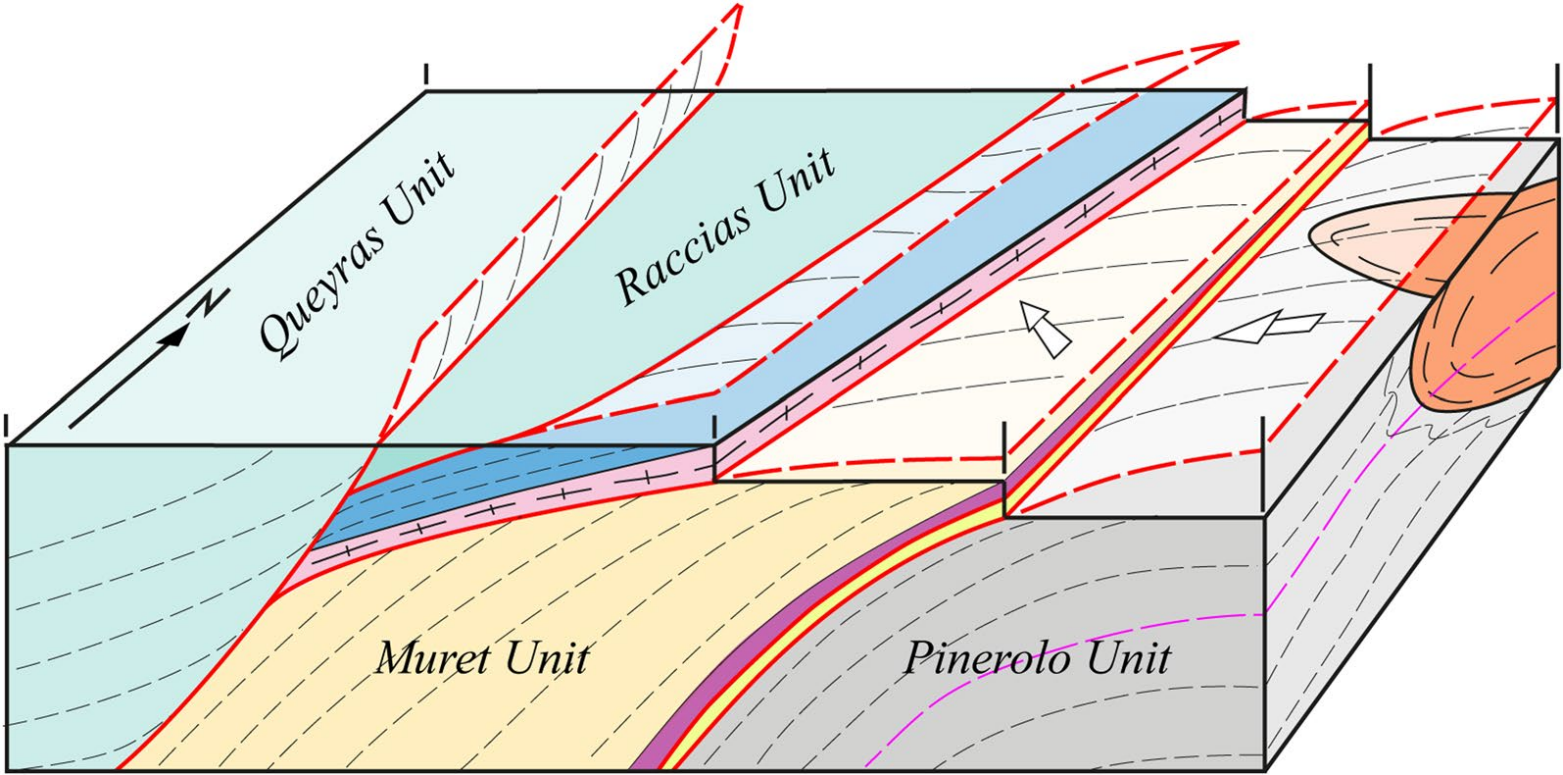
A schematic sketch of the tectonic architecture of the northern Dora-Maira Massif. The units are depicted with the same colors used in Figs. 2 and 3. The yellow thin slice between the Pinerolo and the Muret Units is the Chasteiran Unit

The Alpine tectonic history of the northern Dora-Maira Massif consists of several superposed episodes (Fig. 18). The oldest recognizable stages are associated with the burial of the thinned continental margin in a subduction zone. Witnesses of this stage are, paradoxically, the lack of Mesozoic cover on top of the Pinerolo Unit, because it should have been detached already in the earliest stages of burial. More direct evidence of this burial is recorded by the early growth stages of garnet in the Chasteiran (Manzotti et al., 2022) and Muret Units (Nosenzo et al., 2023). The development of *HP* to *UHP* assemblages is associated, with a few exceptions, to a pervasive ductile deformation (D_1_).

Stacking of the different units from the Dora-Maira Massif has taken place during exhumation, associated to the main, regional, ductile deformation (D_2_), in the albite stability field. The main ductile boundary in the nappe stack is the one separating in the footwall the Pinerolo Unit and in the hangingwall the Muret Unit. The *UHP* Chasteiran Unit is located along this boundary (Fig. 3), in a similar position than the *UHP* Brossasco-Isasca Unit in the southern Dora-Maira Massif (e.g. Henry et al., 1993). In the studied area, the mylonitic deformation of the Granero Orthogneiss is the clearest expression of the tectonic boundary between the Pinerolo and Muret Units, in this case characterized by a top-to-the-West displacement of the hangingwall with respect to the footwall. Details of these two deformation stages (D_1_ and D_2_) need due consideration of the *P‒T‒t* history of the units now in contact, but widely separated in the subduction zone, a topic that requires further geochronological and petrological data, beyond the scope of this paper.

Late reworking of some tectonic boundaries at the ductile to brittle transition has taken place, especially along the western margin of the Dora-Maira Massif. Specifically, the contact between the Muret and Serre Units is marked by a thin (a few metres thick) but marked zone of deformation, with CS structures and asymmetric folds overprinting the D_2_ deformation (Figs. 15, 16, 17). These top-to-the-NW structures may be related to the late deformation along the Vallanta-Susa Shear Zone, a D_3_ structure cutting across and reworking most of the nappe stack along the western margin of the Dora-Maira Massif (Ballèvre et al., 1990; Ghignone et al., 2020).

Finally, late brittle normal faults (i.e. the Trossieri Fault in the study area), cut across the nappe stack as well as the Vallanta-Susa Shear Zone. These late faults are identified inside the Dora-Maira Massif and may be linked to the present seismotectonic frame (e.g. Perrone et al., 2009).

## Conclusions and perspectives

The main conclusions of our field and geochronological investigations in the northern Dora-Maira Massif are as follows (Fig. 18).

1. The lowermost unit, i.e. the Pinerolo Unit, consists of Late Carboniferous sediments deposited in a variety of environments, from fluvial to lacustrine. The sediments have been intruded by granitic and dioritic plutonic bodies. Their putative sedimentary cover has been detached from the downgoing slab at the onset of the Alpine orogenic history.
2. A major ductile shear zone, assumed to be a westward thrust, emplaces the Chasteiran and Muret Units on top of the Pinerolo Unit. The polycyclic basement of the Muret Unit consists of late Neoproterozoic to early Palaeozoic sediments intruded by late Ordovician to early Silurian granitoid bodies. At map scale, we propose that the basal contact of the Muret Unit is best defined by a remarkable reference layer, namely the Granero Orthogneiss.
3. On top of the nappe stack, the Serre Unit, consisting of Permian plutonic, volcanic and volcano-clastic rocks, and a thin Mesozoic cover, is separated from its footwall by a narrow, ductile to brittle, shear zone (La Fracho Shear Zone).
4. The nappe stack is lately affected by normal faulting, indicating a NNW-SSE extensional episode.

Overall, the tectonic stack of the Dora-Maira Massif is consistent from north to south. The metamorphic architecture is analogous in the northern and southern part of the massif, with a *UHP* unit sandwiched between two lower *P* units, the Pinerolo Unit below and a basement unit above. Further investigations in the Pinerolo and Serre Units, will be necessary to evaluate differences of *P‒T* conditions, and their potential link with the tectonic history.

### Supplementary Information

**Additional file 1: Appendix S1.** Operating conditions of LA-ICP-MS equipment for zircon U–Pb analysis. Working conditions for session1 refer to the samples OG34, OG36 and OG9, whereas those for session2 refer to samples OG49, PG3 and PG41, as the samples were analysed on different days. **Appendix S2.** Operating conditions of LA-ICP-MS equipment for zircon trace element analysis. Working conditions for session1 refer to the samples OG34, OG36 and OG9, whereas those for session2 refer to sample OG49, as the samples were analysed on different days. **Figure S1.** Whole-rock geochemistry of the studied orthogneiss (sample OG34, OG36 and OG49, Granero Orthogneiss; sample OG7 and OG9, Clapier Orthogneiss). a R1–R2 classification diagram for plutonic and volcanic rocks (De La Roche et al., 1980). The Muret Orthogneiss (sample OG27, Nosenzo et al., 2022), the Sangone Orthogneiss, the Freidour Orthogneiss, the Cavour Orthogneiss and the Malanaggio Meta-diorite (San., Fre., Cav. and Mal., respectively; Bussy & Cadoppi, 1990) are also plotted for comparison. b Total alkali vs. silica (TAS) classification diagram for volcanic rocks (Le Maitre et al., 2002). **Figure S2.** Concordia diagram for the Granero Orthogneiss. Data from samples OG34, OG36, and OG49 are plotted together. Empty dashed ellipses represent dates excluded from the concordia age calculation, as they are affected by Pb loss or mixing with metamorphic rims. **Table S1.** Bulk-rock compositions of orthogneisses in the northern Dora-Maira Massif.

**Additional file 2: Table S2.** Zircon U–Pb and trace element dataset.

## Data Availability

The online version contains supplementary material available at XXXX (Additional files).
