# Supplementary material for: Tectonic architecture of the northern Dora-Maira Massif (Western Alps, Italy): field and geochronological data
Source: Swiss J Geosci. 2024 Apr 22;117(1):6. doi: 10.1186/s00015-024-00459-2 (PMC11035143; doi:10.1186/s00015-024-00459-2)
Supplement: Supplementary file 1 — Additional file 1: Appendix S1. Operating conditions of LA-ICP-MS equipment for zircon U–Pb analysis. Working conditions for session1 refer to the samples OG34, OG36 and OG9, whereas those for session2 refer to samples OG49, PG3 and PG41, as the samples were analysed on different days. Appendix S2. Operating conditions of LA-ICP-MS equipment for zircon trace element analysis. Working conditions for session1 refer to the samples OG34, OG36 and OG9, whereas those for session2 refer to sample OG49, as the samples were analysed on different days. Figure S1. Whole-rock geochemistry of the studied orthogneiss (sample OG34, OG36 and OG49, Granero Orthogneiss; sample OG7 and OG9, Clapier Orthogneiss). a R1–R2 classification diagram for plutonic and volcanic rocks (De La Roche et al., 1980). The Muret Orthogneiss (sample OG27, Nosenzo et al., 2022), the Sangone Orthogneiss, the Freidour Orthogneiss, the Cavour Orthogneiss and the Malanaggio Meta-diorite (San., Fre., Cav. and Mal., respectively; Bussy & Cadoppi, 1990) are also plotted for comparison. b Total alkali vs. silica (TAS) classification diagram for volcanic rocks (Le Maitre et al., 2002). Figure S2. Concordia diagram for the Granero Orthogneiss. Data from samples OG34, OG36, and OG49 are plotted together. Empty dashed ellipses represent dates excluded from the concordia age calculation, as they are affected by Pb loss or mixing with metamorphic rims. Table S1. Bulk-rock compositions of orthogneisses in the northern Dora-Maira Massif. [file 15_2024_459_MOESM1_ESM.pdf]

Additional file 1

Tectonic architecture of the northern Dora-Maira Massif (Western Alps, Italy): field and geochronological data

Francesco Nosenzo [1], Paola Manzotti [1*], Mikaela Krona [1,3], Michel Ballèvre [2], Marc Poujol [2]

Affiliations

[1] Department of Geological Sciences, Stockholm University, 106 91, Stockholm, Sweden

[2] Géosciences Rennes-UMR 6118, University of Rennes, F35000, Rennes, France

[3] Department of Geosciences, University of Padova, Italy

*Correspondence: paola.manzotti@geo.su.se; ORCID: 0000-0002-6945-9878

Appendix S1 Operating conditions of LA-ICP-MS equipment for zircon U-Pb analysis. Working conditions for session1 refer to the samples OG34, OG36 and OG9, whereas those for session2 refer to samples OG49, PG3 and PG41, as the samples were analysed on different days.

**Laboratory & Sample Preparation**	
Laboratory name	GeOHeLiS Analytical Platform, OSUR, Univ. Rennes 1, France
Sample preparation	Conventional mineral separation at Stockholm University (SU) and at Swedish Museum of Natural History (NRM).
Imaging	Cathode luminescence (CL) using a XL30 ESEM-FEG with Centaurus detector (15 kV voltage) at NRM
**Laser ablation system**	
Make, Model & type	ESI NWR193UC, Excimer
Ablation cell	ESI NWR TwoVol2
Laser wavelength	193 nm
Pulse width	< 5 ns
Fluence	6.4 J/cm^2 (session1), 6.6 J/cm^2 (session2)
Repetition rate	4Hz
Spot size	25 µm
Sampling mode / pattern	Single spot
Carrier gas	100% He, Ar make-up gas and N2 (3 ml/mn) combined using in-house smoothing device
Background collection	20 seconds
Ablation duration	60 seconds (session1), 40 seconds (session2)
Wash-out delay	15 seconds
Cell carrier gas flow (He)	0.76 l/min
**ICP-MS Instrument**	
Make, Model & type	Agilent 7700x, Q-ICP-MS
Sample introduction	Via conventional tubing
RF power	1350W
Sampler, skimmer cones	Ni
Extraction lenses	X type
Make-up gas flow (Ar)	0.70 l/min (session1), 0.67 l/min (session2)
Detection system	Single collector secondary electron multiplier
Data acquisition protocol	Time-resolved analysis
Scanning mode	Peak hopping, one point per peak
Detector mode	Pulse counting, dead time correction applied, and analog mode when signal intensity > ~ 10^6 cps
Masses measured	^{204}(Hg + Pb), ^{206}Pb, ^{207}Pb, ^{208}Pb, ^{232}Th, ^{238}U
Integration time per peak	10-30 ms (^{207}Pb)
Sensitivity / Efficiency	23000 cps/ppm Pb (session1), 23000 cps/ppm Pb (session2) (50µm, 10Hz)

**Data Processing**	
Gas blank	20 seconds on-peak
Calibration strategy	GJ1 zircon standard used as primary reference material, Plešovice used as secondary reference material (quality control)
Common-Pb correction, composition and uncertainty	No common-Pb correction.
Reference Material info	GJ1 (Jackson et al., 2004), Plešovice (Sláma et al., 2008)
Data processing package	Iolite (Paton et al., 2010)
Uncertainty level and propagation	Individual dates are quoted at 2σ absolute, whereas concordia ages are given with 95% confidence level. Propagation is by quadratic addition according to Horstwood et al. (2016). Reproducibility and age uncertainty of reference material are propagated.
Quality control / Validation	Plešovice: concordia age = 336.9± 1.7 Ma (N=16; MSWD=1.0; session1), 336.8± 1.1 Ma (N=48; MSWD=0.74; session2)

Appendix S2 Operating conditions of LA-ICP-MS equipment for zircon trace element analysis. Working conditions for session1 refer to the samples OG34, OG36 and OG9, whereas those for session2 refer to sample OG49, as the samples were analysed on different days.

**Laboratory & Sample Preparation**	
Laboratory name	GeOHeLiS analytical Platform, Université Rennes 1, France
Sample preparation	Conventional mineral separation at Stockholm University (SU) and at Swedish Museum of Natural History (NRM).
**Laser ablation system**	
Make, Model & type	ESI NWR193UC, Excimer
Ablation cell	ESI NWR TwoVol2
Laser wavelength	193 nm
Pulse width	< 5 ns
Fluence	6.4 J/cm^2 (session1), 6.6 J/cm^2 (session2)
Repetition rate	4 Hz
Spot size	25 µm
Sampling mode / pattern	Single spot
Carrier gas	100% He, Ar make-up gas and N_2 (3 ml/mn) combined using in-house smoothing device
Background collection	20 seconds
Ablation duration	60 seconds
Wash-out delay	15 seconds
Cell carrier gas flow (He)	0.76 l/min
**ICP-MS Instrument**	
Make, Model & type	Agilent 7700x, Q-ICP-MS
Sample introduction	Via conventional tubing
RF power	1350W
Sampler, skimmer cones	Ni
Extraction lenses	X type
Make-up gas flow (Ar)	0.68 l/min (session1), 0.67 l/min (session2)
Detection system	Single collector secondary electron multiplier
Data acquisition protocol	Time-resolved analysis
Scanning mode	Peak hopping, one point per peak
Detector mode	Pulse counting, dead time correction applied, and analog mode when signal intensity > ~ 10^6 cps
Masses measured	^{29}Si, ^{31}P, ^{49}Ti, ^{88}Sr, ^{89}Y, ^{93}Nb, ^{139}La, ^{140}Ce, ^{141}Pr, ^{146}Nd ^{147}Sm, ^{153}Eu, ^{157}Gd, ^{159}Tb, ^{163}Dy, ^{165}Ho, ^{166}Er, ^{169}Tm, ^{172}Yb, ^{175}Lu, ^{178}Hf, ^{181}Ta, ^{204}(Hg + Pb), ^{206}Pb, ^{207}Pb, ^{208}Pb, ^{232}Th, ^{238}U
Integration time per peak	10 ms (except for ^{207}Pb 30 ms)
Total integration time per reading	340 ms (session1), 260 ms (session2)
Sensitivity / Efficiency	21000 cps/ppm Pb (session1), 22000 cps/ppl Pb (session 2) (50µm, 10Hz)
**Data Processing**	
Gas blank	20 seconds on-peak
Calibration strategy	NIST 612 as primary reference materials, 91500 zircon standard as secondary reference material (quality control)
Reference Material info	NIST 612<br>91500 (Wiedenbeck et al., 1995; 2004)
Data processing package used	Iolite (Paton et al., 2010)

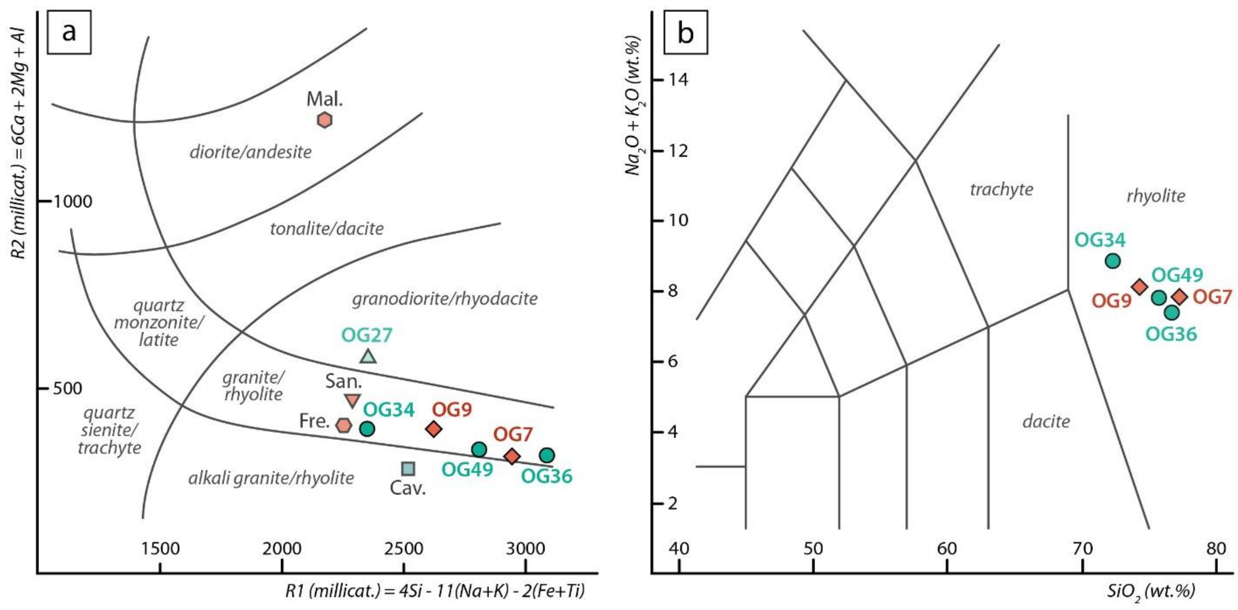

Fig. S1 Whole-rock geochemistry of the studied orthogneiss (sample OG34, OG36 and OG49, Granero Orthogneiss; sample OG7 and OG9, Clapier Orthogneiss). **a** R1-R2 classification diagram for plutonic and volcanic rocks (De La Roche et al., 1980). The Muret Orthogneiss (sample OG27, Nosenzo et al., 2022), the Sangone Orthogneiss, the Freidour Orthogneiss, the Cavour Orthogneiss and the Malanaggio Meta-diorite (San., Fre., Cav. and Mal., respectively; Bussy & Cadoppi, 1990) are also plotted for comparison. **b** Total alkali vs. silica (TAS) classification diagram for volcanic rocks (Le Maitre et al., 2002).

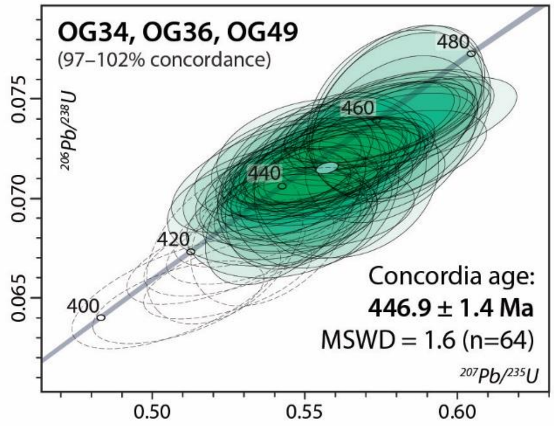

Fig. S2 Concordia diagram for the Granero orthogneiss. Data from samples OG34, OG36, and Og49 are plotted together. Empty dashed ellipses represent dates excluded from the concordia age calculation, as they are affected by Pb loss or mixing with metamorphic rims.

wt%	Sample	SiO_2	Al_2O_3	CaO	MgO	MnO	P_2O_5	Fe_2O_3	Na_2O	K_2O	TiO_2	NK	A/CNK (mol)
Granero orthogneiss	OG34	72.32	14.89	0.62	0.60	0.03	0.17	2.25	2.70	6.17	0.26	8.87	1.22
	OG36	76.71	13.01	0.40	0.40	0.02	0.17	1.73	1.90	5.52	0.13	7.43	1.32
	OG49	75.69	13.41	0.61	0.22	0.03	0.18	1.86	2.92	4.94	0.14	7.86	1.19
Muret orthogneiss (Nosenzo et al., 2022)	OG27	67.10	15.74	1.79	1.58	0.09	0.21	5.73	2.65	4.30	0.82	6.95	1.28
Clapier orthogneiss	OG7	77.16	12.77	0.38	0.40	0.01	0.15	1.18	2.70	5.15	0.10	7.85	1.19
	OG9	74.34	13.85	0.88	0.40	0.05	0.08	2.03	3.00	5.15	0.21	8.15	1.14

Table S1 Bulk-rock compositions of orthogneisses in the northern Dora-Maira Massif.

References

De La Roche, H., Leterrier, J., Grandclaude, P., & Marchal, M. (1980). A classification of volcanic and plutonic rocks using R_1R_2-diagram and major element analyses – Its relationships with current nomenclature. Chemical Geology, 29, 183-210.

Jackson, S.E., Pearson, N.J., Griffin, W.L., & Belousova, E.A. (2004). The application of laser ablation-inductively coupled plasma-mass spectrometry to in situ U–Pb zircon geochronology. Chemical Geology, 211, 47-69.

Horstwood, M.S.A., Košler, J., Gehrels, G., Jackson, S.E., Mc, L.N., Paton, C., Pearson, N.J., Sircombe, K., Sylvester, P., Vermeesch, P., Bowring, J.F., Condon, D.J., & Schoene, B. (2016). Community-derived standards for LA-ICP-MS U-(Th-) Pb geochronology—Uncertainty propagation, age interpretation and data reporting. Geostandards and Geoanalytical Research, 40, 311-332.

Le Maitre, R.W. (2002). Igneous rocks. A classification and glossary of terms. Cambridge: Cambridge University Press.

Paton, C., Woodhead, J.D., Hellstrom, J.C., Herget, J.M., Greig, A., & Maas, R. (2010). Improved laser ablation U-Pb zircon geochronology through robust downhole fractionationcorrection. Geochemistry, Geophysics, Geosystems, 11(3), Q0AA06.

Sláma, J., Košler, J., Condon, D.J., Crowley, J.L., Gerdes, A., Hanchar, J.M., … Whitehouse, J.M. (2008). Plesovice zircon—A new natural reference material for U-Pb and Hf isotopic microanalysis. Chemical Geology, 249, 1-35.

Wiedenbeck, M., Allé, P., Corfu, F., Griffin, W.L., Meier, M., Oberli, F., Von Quadt, A., Roddick, J.C., & Spiegel, W. (1995). Three natural zircon standards for U-Th-Pb, Lu-Hf, trace element and REE analyses. Geostandards Newsletter, 19(1), 1-23.

Wiedenbeck, M., Hanchar, J.M., Peck, W.H., Sylvester, P., Valley, J., Whitehouse, M., … & Zheng, Q. Y.-F. (2004). Further Characterisation of the 91500 Zircon Crystal. Geostandards and Geoanalytical Research, 28(1), 9-39.
